# Novel Alginate-Based Physical Hydrogels: Promising Cleaning Tools for Sensitive Artifacts

**DOI:** 10.3390/polym17222976

**Published:** 2025-11-08

**Authors:** Matteo Ferretti, Maduka L. Weththimuni, Donatella Sacchi, Chiara Milanese, Alessandro Girella, Barbara Vigani, Gaia Zucca, Alice Pedalà, Nicola Razza, Maurizio Licchelli

**Affiliations:** 1 Department of Chemistry, Università di Pavia, 27100 Pavia, Italy; matteo.ferretti02@universitadipavia.it (M.F.); donatella.sacchi@unipv.it (D.S.); chiara.milanese@unipv.it (C.M.);; 2 Research Centre for Cultural Heritage (CISRiC), Università di Pavia, 27100 Pavia, Italy; alice.pedala01@universitadipavia.it (A.P.); nicola.razza01@universitadipavia.it (N.R.); 3 Department of Drug Science, Università di Pavia, 27100 Pavia, Italy

**Keywords:** polysaccharides, hydrogels, shape control, alginate, physical crosslinking, artifact cleaning, wood materials, iron gall ink, SEM-EDX, FT-IR

## Abstract

Natural polysaccharides are used for very different applications and are particularly exploited for preparing hydrogel materials. For instance, gels based on different carbohydrate polymers have been applied to remove unwanted materials from the surface of cultural heritages items. This study was focused on the preparation of novel physical hydrogels suitable for the cleaning of sensitive materials like wood and paper, i.e., to remove the soil from their surface. For this purpose, alginate biopolymer was used and ionically crosslinked with six different amines, in the presence of N-hydroxysuccinimide as a co-gelling agent. All the synthetized gel materials were characterized by a multianalytical approach, using different techniques such as FT-IR, thermal analysis, SEM-EDS, mechanical tests, and evaluation of moisture properties. All the results showed that the introduction of the investigated amines improved the original properties of alginate and provided good cleaning properties when applied to sensitive surfaces.

## 1. Introduction

Natural carbohydrate polymers are attracting growing interest as building blocks for the development of new materials that can meet requirement of sustainability, environmental safety, and durability at the same time. Among their properties, the tendency to form gels is widely investigated, as gel-based materials find applications across a variety of fields, including food and pharmaceutical industries, biomedicine, environmental engineering, and the conservation of cultural heritage [1,2,3,4,5,6,7,8]. For instance, some gels have been studied even as carriers for active molecules, such as drugs in pharmaceutical formulations [9,10].

Gel materials, which can be generally obtained by dispersing polymers in solvents, typically water or water-based mixtures (e.g., emulsions), can be divided into two main families, depending on the nature of the interactions that hold the structure together: physical gels, where polymer chains are crosslinked via reversible non-covalent interactions (e.g., ionic, hydrogen bonding), and chemical gels, in which covalent bonds provide the structural framework [11,12,13,14].

Over the past decade, different natural polymers, such as alginate, agar, gellan gum, xanthan gum, chitosan, and their derivatives, have all been used to produce hydrogels, where water forms the main or only liquid phase [15,16,17,18,19].

In this context, and as part of our broader work on developing innovative gels for conservation purposes [20,21,22], we present here the preparation and study of novel physically crosslinked gels based on alginate polymer and polyammonium ions.

**Figure 1:**
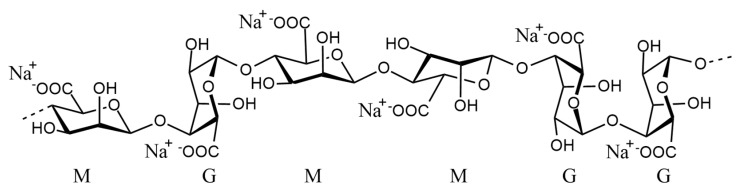
Structure of sodium alginate (M: mannuronic acid; G: guluronic acid).

Alginate is a linear polysaccharide derived from brown algae and some bacterial species. It is composed of β-D-mannuronic acid (M) and α-L-guluronic acid (G) residues linked through 1,4-glycosidic bonds (Figure 1). Commercially available as water-soluble sodium alginate, the ratio between G and M blocks influences the gel properties: M-rich regions tend to be more elastic, while G-rich domains provide greater stiffness. The sequence and length of these blocks play a crucial role in determining the mechanical and functional behavior of the material [23,24,25].

Thanks to its biocompatibility, non-toxicity, and biodegradability, alginate has been widely used in food and cosmetic formulations. In the biomedical field, alginate is valued for its mucoadhesive properties, which can improve drug delivery by increasing the residence time at the site of action and enhancing both efficacy and bioavailability [26,27,28]. In food technology, alginate is applied as a coating for fruits and vegetables protecting them from microbial contamination and serves as a gelling, thickening, stabilizing, or emulsifying agent [29,30,31].

More recently, alginate-based materials have been studied as adsorbents for removing environmental contaminants from different matrices [32,33,34]. In the field of cultural heritage preservation, alginate hydrogels are mostly used to clean surfaces by removing dirt, dust, and degraded materials such as old varnishes or adhesives. Their ability to conform to curved or delicate surfaces makes them particularly versatile and gentle tools for conservation professionals [32,35,36].

Alginate hydrogels are usually formed in the presence of divalent or trivalent metal ions such as Ba^2+^, Sr^2+^, Ca^2+^, Mg^2+^, and Al^3+^, through electrostatic interactions with its carboxylate groups. Gelation is particularly favored by spherical divalent cations, which promote the self-assembly of the polymeric structure. In contrast, monovalent cations are generally insufficient to induce crosslinking [23,24,37,38]. Since these interactions are electrostatic in nature, the gelation process is highly sensitive to experimental and environmental parameters, such as pH, temperature, the type and source of the salt used, and its concentration [39,40,41].

Although alginate hydrogels offer many advantages, they tend to suffer from poor mechanical strength and are susceptible to microbial degradation. These limitations have prompted efforts within the scientific community to develop modified or improved materials.

**Figure 2:**
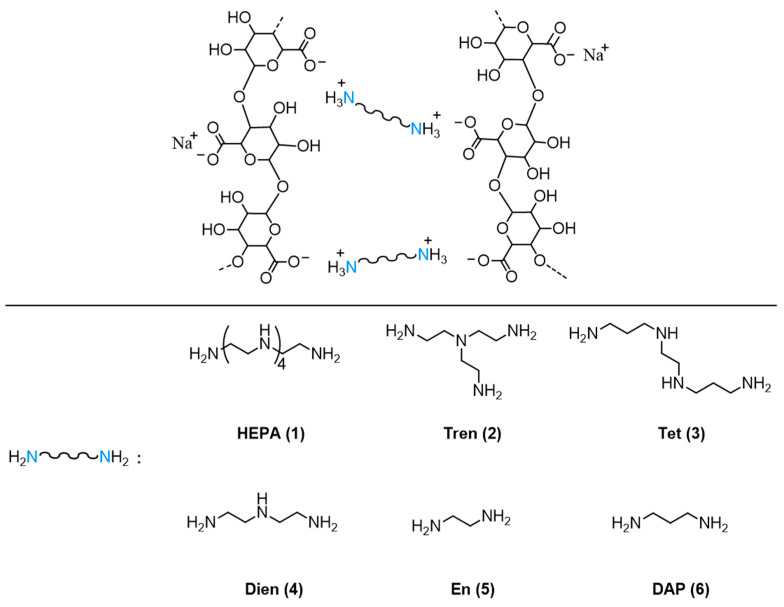
Schematic crosslinking of alginate polymer chains by different aliphatic protonated polyamines.

In the present work, we describe the preparation of novel alginate-based hydrogels formed without the use of calcium salts. In this case, gelation is partially driven by aliphatic polyamines and their corresponding ammonium ions, which induce physical crosslinking of the polymer chains (Figure 2) in the presence of N-hydroxysuccinimide, which acts as a co-gelling agent. This strategy aims to exploit the gelation effect induced by polyammonium species as well as their antifungal properties.

In fact, ammonium ions and related amines have broad-spectrum antimicrobial properties, including antifungal activity [42,43,44,45] and are widely used across industrial, medical, and environmental sectors as disinfectants and preservatives [46]. In the cultural heritage field, ammonium-based substances are of particular interest for surface cleaning, especially on sensitive artworks and materials [47]. Their antimicrobial activity is due to several mechanisms: they disrupt microbial membranes [48], inhibit key metabolic functions [49], and block biofilm formation, which is one of the main survival strategies of microbes [50,51,52,53].

Although free ammonium compounds can sometimes pose health or environmental risks, incorporating them into polymer-based gels such as those made with natural alginate may offer a smart way to boost material durability against microorganism attacks, while minimizing potential hazards.

The investigated materials obtained from the combination of alginate polymer and different polyamine/ammonium compounds have been characterized through a multianalytical approach, which showed that the hydrogel properties (e.g., mechanical performance, shape retention, and modulated water-related behavior) can be modulated depending on the envisaged polyamine. Furthermore, the resulting gels loaded with different water/solvent mixtures have been tested as cleaning tools for the removal of unwanted materials from the surface of wood and paper-based supports. Preliminary results are suggestive of good cleaning effectiveness and are very promising in view of possible applications in the field of cultural heritage conservation [54].

## 2. Experimental Sections

### 2.1. Materials

Sodium alginate (SA, Alginic acid sodium salt from brown algae, low viscosity); 3-(N-morpholino)propanesulfonic acid (MOPS, ≥99.5%); N-Hydroxysuccinimide (NHS, ≥97.0%); Tris(2-aminoethyl)amine (Tren, 96%); 3,6,9,12-Tetraazatetradecane-1,14-diamine (Pentaethylenehexamine, PEHA, 80–90%,); 1,2-Bis(3-aminopropylamino)ethane (3,2,3-tet, 94%); Dietilentriammina (Dien, purity 99%); 1,3-Diaminopropane (DAP, purity ≥ 99.0%); and 1,2-Diaminoetane (en, purity ≥ 99.0%) were purchased from Sigma-Aldrich (St. Louis, MO, USA) and used as received. The solvents used for the cleaning tests, i.e., Ethanol (≥99.5%, ACS reagent), 1-butanol (99%, ACS reagent), 1-butanol (≥99.4%, ACS reagent), butanone (≥98%, ACS reagent), and Ecosurf^®^ EH-6 non-ionic surfactant, were purchased from Sigma-Aldrich (St. Louis, MO, USA).

Iron gall ink was prepared according to a literature protocol [55], ensuring the reproducibility of the chemical–physical and chromatic properties typical of historical inks. The reagents used for its preparation included gallnuts and arabic gum, purchased from Kremer Pigmente (Germany, Aichstetten), while iron (II) sulfate heptahydrate (FeSO_4_·7H_2_O) and copper (II) sulfate pentahydrate (CuSO_4_·5H_2_O) were obtained from Sigma-Aldrich (St. Louis, MO, USA). A fortified wine was also included in the ink formulation, selected according to the original Stradivari’s recipe and sourced from a local winery.

Maple wood specimens finished with dewaxed shellac were prepared as previously reported [56,57].

Paper mock-ups were prepared from Whatman No. 1 purified cellulose filter paper. The iron gall ink (100 µL) was deposited onto the paper using a micropipette to ensure reproducible applications. A 2 × 2 cm area of paper was selected, and the ink was spread over the surface with the pipette tip, so that all prepared mock-ups contained the same amount of ink on the surface. The samples were then air-dried for 2 days before use.

The artificial dust was formulated according to a literature procedure [58] with the aim of reproducing deposition conditions relevant to artifacts exposed outdoors. Its composition includes carbon, iron oxide, silica, calcium hydroxide, and kaolin, in order to mimic the inorganic fraction of airborne particulate matter, while starch and gelatin were added to simulate organic residues of animal or anthropogenic origin. This mixture was suspended with chloroform and paraffin oil and applied onto the surface of wood and ink/paper mock-ups using a brush. For the mock-ups employed in this study, the deposition was repeated three consecutive times, allowing complete solvent evaporation between applications.

### 2.2. Crosslinking Procedure

MOPS (2.10 g, 0.01 mol) and sodium alginate (1.00 g, 5.1 × 10^−3^ mol calculated as mol of monomer unit) were mixed in a 250 mL round-bottom flask with water (100 mL). The mixture was mechanically stirred for 12 h (until solubilization of the solid) and pH was measured (pH = 6.5). Then NHS (0.50 g, 4.3 × 10^−3^ mol) was added, and the resulting mixture was stirred for 2 h before adding a solution of HEPA (1.97 g, 5.52 × 10^−3^ mol) in water (15 mL). Stirring was continued for further 12 h; then isopropanol (100 mL) was added and stirring was stopped. The precipitate was filtered on Buckner funnel, washed two times with isopropanol, in order to remove excess reagents, and dried under reduced pressure at r. t. for 12 h. The solid product obtained from the reaction was pounded with a mortar until powder consistence was obtained and stored.

Crosslinking by using other polyamines than HEPA was carried out by the same procedure, taking the same molar ratios.

The products obtained from the polyamines HEPA, Tren, Tet, Dien, En, and DAP were labeled as Alg-1, Alg-2, Alg-3, Alg-4, Alg-5, and Alg-6, respectively.

### 2.3. Preparation of Hydrogel Samples

The hydrogel preparation was optimized with qualitative tests. Silicone molds were used to control the shape during the freezing cycle. Hydrogel samples with two different shapes (film and “rough cube”) were obtained. Hydrogel films were prepared according to the following procedure: polymer (0.3 g) was ground in a mortar and poured in a 20 mL vial with demineralized water (10 mL). The sealed vial was magnetically stirred at 50 °C for 16 h. After this period, the solution was poured into a silicone mold (about 5 × 8 cm^2^) and placed in a freezer at −20 °C for 2 h. Then, the frozen polymer was removed from the mold and placed on Japanese paper until it was dry (approximately 16 h). The dehydrated film can be stored in a refrigerator. To obtain the final hydrogel sample, the dried film was treated with demineralized water (5 mL) and, after about 1 min, the hydrated film was ready for use (rough sizes: 50 mm × 80 mm × 2–3 mm height).

Roughly cubic hydrogel samples (approximately 1 × 1 × 1 cm) were prepared as follows: powdered polymer (0.500 g) was mixed with demineralized water (10 mL) in a 20 mL double-septum vial. Then, the sealed vial was magnetically stirred at 50 °C for 16 h using a sand bath (ensuring the solvent line remains below the sand surface). After this period, the polymer solution was set into a silicone mold (1 × 1 × 1 cm). While the polymer was still warm, it was placed in a freezer at −20 °C for 16 h. After the freezing cycle, the frozen polymer was removed from the mold and exposed to air on Japanese paper until fully dehydrated (approximately 16 h at 20 °C). The dried cube can be stored in a refrigerator. Before use, the cube must be rehydrated in an aqueous solution for 16 h. Excess surface water is removed using Japanese paper.

### 2.4. Characterization of Polymers and Gel Materials

Differential Scanning Calorimetry (DSC): DSC measurements were carried out with a Q2000 calorimeter (TA Instruments, software thermal advantage rel. 5.5, New Castle, DE, USA) under N_2_ flux in Al open crucibles. Prior to analysis, samples were dehydrated under vacuum at room temperature for 24 h. The thermal protocol consisted of the following: (i) heating from 18 to 40 °C at 2 °C/min; (ii) an isothermal step at 40 °C for 30 min to remove residual atmospheric moisture; (iii) cooling from 40 to 16 °C at 5 °C/min; (iv) an isothermal stage at 16 °C for 60 min to stabilize the material; and (v) heating from 16 to 65 °C at 2 °C/min. The dehydration was deliberately limited to T ≤ 40 °C to prevent material degradation. Indeed, attempts performed at higher temperatures (above T_g_) led to partial deterioration, as indicated by a brownish discoloration of the polymers. Three independent measurements were taken on each sample. The temperature accuracy of the instrument is ±0.1 °C, the precision is ±0.01 °C, and the calorimetric reproducibility is ±0.05%. DSC data were analyzed by the Universal Analysis 2000 v 4.5 software by TA Instruments.

Thermogravimetric analysis (TGA): Thermogravimetric analysis (TGA) was performed by a Q5000 apparatus (TA Instruments, New Castle, DE, USA) interfaced with a TA5000 data station under nitrogen flux (10 mL min^−1^) in a platinum pan by heating about 5 mg of sample from room temperature up to 220 °C (heating rate 5 K min^−1^). TGA data were analyzed by the Universal Analysis 2000 v 4.5 software by TA Instruments.

Fourier-Transform Infrared (FT-IR) spectroscopy: Infrared spectra were acquired using a PerkinElmer Spectrum 100 FT-IR spectrometer (PerkinElmer, Waltham, MA, USA) equipped with a diamond ATR accessory. Samples were dried before measurement for 24 h under vacuum at room temperature in the presence of silica gel as a desiccant. The spectra were recorded at 25 °C with 64 scans, in the range from 4000 to 550 cm^−1^.

SEM/EDS analysis: Morphological and elemental analyses were performed by scanning electron microscopy (SEM, backscattered electron mode) coupled with energy dispersive spectroscopy (EDS) using a Tescan FE-SEM MIRA 3XMU (Tescan, Brno, Czech Republic), equipped with a Schottky field emission source. Thin polymer films were preliminarily dehydrated at 25 ± 2 °C for 16 h, ensuring long-term stability. Before SEM/EDS acquisition, the films were further dried in a vacuum desiccator for 30 min (using a gentle vacuum to avoid damage), and subsequently sputtered with graphite using a Cressington Carbon Coater 208 C.

Moisture-related properties: The equilibrium water content (EWC%) was quantified by gravimetric analysis as reported in Equation (1). Gel samples were weighed before and after immersion in distilled water at 25 ± 2 °C for 5 days, until constant weight was achieved:

EWC% = (W_w_ − W_d_)/W_w_ × 100 (1)

where W_d_ is the dry weight of the gel, and W_w_ is the hydrated weight.

The retention capacity (RC) was determined under the same temperature conditions, according to Equation (2). Dried samples were hydrated with distilled water for 30 min and then transferred onto filter paper for 20 min in a closed plastic container. The weight difference of the filter paper before and after contact with the gel was measured:

RC = (F_w_ − F_d_)/A (2)

where F_d_ and F_w_ are the weights of the dry and wet filter paper, respectively, and A is the contact area.

Mechanical testing: Mechanical properties were evaluated on hydrated polymer samples, either as thin films or cubic gels. Tensile tests were carried out on rectangular films (1 × 3 cm^2^) using a TA.XT plus Texture Analyzer (Stable Micro Systems, Godalming, UK) with a 5 kg load cell and A/TG tensile grips. The initial distance between grips was set to 1 cm, and the upper grip was raised at 2 mm/min over a 50 mm displacement. Film thickness was measured with a Sicutool 3955G-50 (Milan, Italy). Tensile strength and elongation at break were derived, with at least three replicates per film. Compression tests were performed on cubic samples (≈1 × 1 × 1 cm^3^) using a P/10 cylindrical probe (10 mm diameter), applied at a constant speed of 1.00 mm/s until 70% deformation was reached. Five replicates were tested for each cube. Hardness was calculated from the maximum compressive force (Fmax) normalized to the contact area.

The viscoelastic properties of the hydrogels were evaluated using a dynamic mechanical rheometer (Anton Paar MCR 702e, Rivoli, TO, Italy) equipped with a parallel-plate geometry (25 mm diameter) operating in compression mode. Samples were positioned between the plates, and a normal preload of 0.05 N was applied to ensure proper contact without inducing deformation. Measurements were performed at 25 °C, and samples were kept hydrated throughout testing. An amplitude sweep test was first conducted at a fixed angular frequency of 0.5 Hz over a shear stress range of 10^−5^–1 MPa to determine the linear viscoelastic region (LVR) and the yield point. The yield point was defined as the stress at which the storage modulus deviated by 10% from its plateau value. A frequency sweep test was subsequently performed at the shear stress at 2/3 of the LVR, over the range 0.5–10 Hz, for each sample. Extensional storage modulus (E′), extensional loss modulus (E″), and extensional loss factor (tan δ) were recorded to characterize the viscoelastic behavior of the gels. The loss factor was calculated as the ratio E″/E′. Values of tan δ < 1 indicate a predominantly elastic response, whereas tan δ > 1 denotes a predominantly viscous behavior. All experiments were performed on three different samples, and results are reported as mean ± standard deviation.

### 2.5. Resistance to Fungal Attack

To evaluate the resistance of the materials to fungal growth, the Alg-Ca (reference), Alg-1, and Alg-2 samples were exposed at 22 ± 2 °C maintained and continuously hydrated by periodically spraying demineralized water [59]. The gels were maintained in controlled conditions for fifteen days, during which fungal microorganisms were allowed to grow, according to the literature [45]. At the end of the exposition time, the samples were examined by optical microscope (OM, Nikon Eclipse ME600L, Halogen Lamp 12 V 100 W model C-LP, Nikon Instruments S.p.A., Florence, Italy) and SEM, in order to monitor the possible development of fungal colonies on their surfaces.

### 2.6. Preliminary Cleaning Tests

For the removal of artificial dust from mock-ups, the following general procedure was adopted.

Portions of gel film corresponding to predefined areas on the surface of the mock-ups were cut when the gels were completely dry. Before application, gel films were hydrated in deionized water for variable times (commonly 5–10 min, depending on their moisture properties, i.e., EWC%) or loaded with the different cleaning mixture selected for the cleaning tests (1-butanol/water 1:9 *v*/*v*, ethanol/water 1:9 *v*/*v*, 1% Ecosurf^®^ in water, and nanoemulsion composed of 9.7% 1-butanol, 20.9% butanone, and 3.5% Ecosurf^®^ in water [60]). Cleaning tests using gels loaded with plain demineralized water were performed for comparison [61].

Cleaning was carried out by applying the hydrated gel to the selected area and gently promoting its adhesion to the surface, in order to allow a gradual release of water. After 5 min of contact, 10 µL of solvent mixture was further deposited on the surface of the gel using a micropipette, followed by an additional 5 min of contact. This procedure was repeated twice, for a total application time of 15 min.

μ-FT-IR spectroscopic measurements on the surface of wood and paper mock-ups were carried out using a Thermo Scientific Nicolet iN10 MX instrument (ThermoFisher Scientific, Waltham, MA, USA) at 25 °C, operating in ATR (Attenuated Total Reflectance) mode. Colorimetric analyses were performed at 25 °C on each mock-up, selecting three measurement points within the selected test area. The measurements were performed using a Konica Minolta CM-2600d colorimeter (Konica Minolta, Inc., Tokyo, Japan), with an aperture size of 8 mm. For each point, L*, a*, and b* coordinates of the CIELab space were considered and the overall chromatic variations were expressed as ∆E* according to the Formula (3) [62,63,64].

(3) $$\Delta E^{*}=\sqrt{{\left(\Delta L^{*}\right)}^{2}+{\left(\Delta a^{*}\right)}^{2}+{\left(\Delta b^{*}\right)}^{2}}$$

Optical microscope (OM) observations were performed by an Olympus BX51TF microscope equipped with the Olympus TH4-200 lamp (Olympus Corporation, Tokyo, Japan).

## 3. Results and Discussion

### 3.1. Gelling Process and FT-IR Analysis

The synthesis of alginate-based gels by using divalent cations, which induce the formation of stable gel networks, has been widely investigated in the literature [23,24,37,38,65]. In the present study, the crosslinking of alginate chains was carried out by mimicking the role of divalent cations such as calcium, using two (or more) ammonium groups obtained by protonation of aliphatic di- or poly-amine species (compounds 1–6 in Figure 2). The aim was to investigate how such cationic systems can modulate the properties of the resulting gels. The crosslinking reaction was performed at approximately pH 7, controlled by the addition of MOPS buffer, in the presence of N-hydroxysuccinimide acting as co-gelling agents. MOPS is a zwitterionic compound with a buffering range between pH 6.5 and 7.9, which is highly water-soluble. It should be noted that, when only NHS was added to alginate, no gelling process was observed, suggesting that it is not capable on its own of inducing an effective crosslinking, although it can interact with polymers through hydrogen bonding. In addition, the hydrogel materials obtained solely from polyamine and alginate at pH 7 did not display satisfactory behavior, particularly in terms of mechanical properties and shape control of bulk samples. Therefore, it can be assumed that protonated polyamines and NHS synergically act to induce crosslinking of alginate through both ionic and H-bonding interactions.

**Figure 3:**
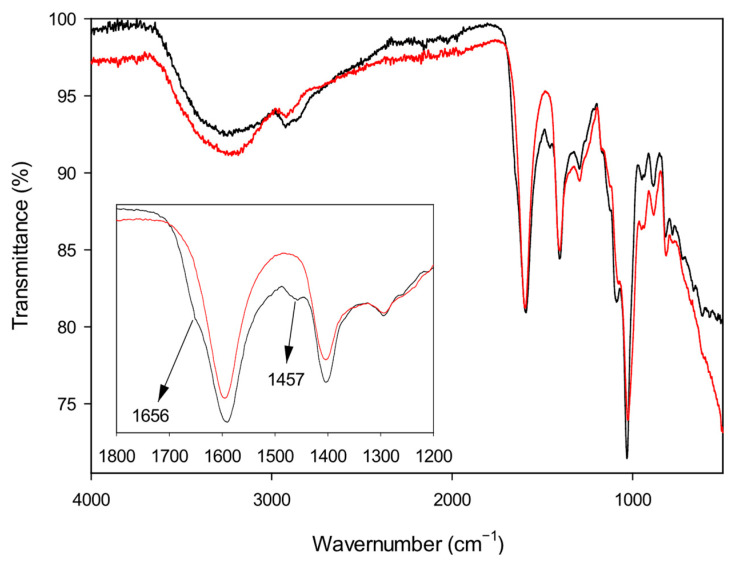
FT-IR spectra of sodium alginate (red) and Alg-1 (black), with a magnified view of the spectral region between 1800 and 1200 cm^−1^.

The interaction between alginate and the gelling agents was first investigated by infrared spectroscopy. FT-IR spectra were recorded on vacuum-dried solid samples and compared with the spectrum of plain alginate, taken as a reference. As an example, the spectra of Alg-1 and Alg-Na are reported in Figure 3. Native sodium alginate shows a broad band at 3426 cm^−1^ attributed to O–H stretching vibration (hydrogen bonding), a signal at about 2930 cm^−1^ assigned to C–H stretching and a strong band at 1592 cm^−1^ due to asymmetric COO^−^ stretching. Additional peaks at 1415 and 1315 cm^−1^ can be attributed to C–OH deformation (with contributions from the symmetric O–C–O stretching of the carboxylic group) and to C–C–H (and O–C–H) deformation, respectively. The bands at 1090 and 1033 cm^−1^ can be assigned to C–O stretching and/or C–C stretching vibrations of pyranose rings [66]. All synthesized products show the main characteristic peaks of sodium alginate, although some small but significant differences can be observed. In particular, the shoulder on the strong carbonyl peak that appears at 1656 cm^−1^ can be due to the carboxylate counterion exchange (from sodium to the ammonium ion). Moreover, the small peak at 1457 cm^−1^, which is absent in the spectrum of pure alginate, can be attributed to the C–H bending of aliphatic polyamines. The individual spectra of all the investigated materials (Alg-1–Alg-6) and of Alg-Na are reported in Figures S1–S7.

### 3.2. Characterization of Modified Alginate: DSC-TGA and SEM-EDS Experiments

**Figure 4:**
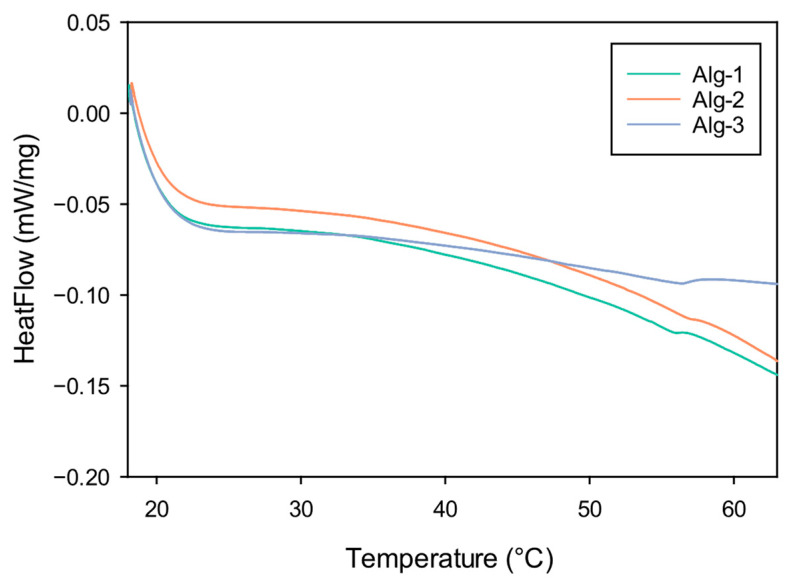
DSC graphs show the glass transitions of the materials Alg-1 (green), Alg-2 (red) and Alg-3 (blue).

Differential Scanning Calorimetry (DSC) experiments on the investigated materials were first performed below 65 °C to prevent possible degradation processes. The calorimetric profiles obtained by DSC analyses performed on samples of dehydrated Alg-1, Alg-2, and Alg-3 are reported in Figure 4. DSC measurements were performed also on powders of polymers Alg-4, Alg-5, and Alg-6, although they are poorly capable of forming gels. The single colorimetric profiles corresponding to all the investigated materials are reported in Figures S8–S13. All materials undergo a glass transition process at about 55–57 °C (Table 1).

The T_g_ values observed for the modified materials are distinctly higher than native Alg-Na, for which a transition temperature of 37.5 °C was reported [67].

This finding strongly supports the hypothesis that a crosslinking process occurs between polysaccharide chains and aliphatic amines, mediated by ionic and H-bond interactions involving carboxylate groups of alginates, ammonium groups of protonated polyamines, and NHS. Furthermore, only a single transition process was observed in the calorimetric profiles of the modified polymers. The absence of the transition corresponding to plain alginate (around 37 °C) indicates that the structural changes of the materials are quite homogeneous.

**Figure 5:**
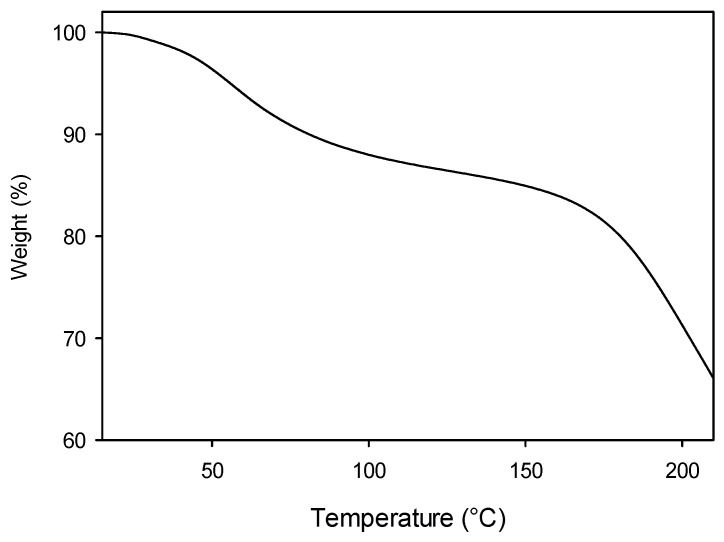
Thermogravimetric analysis (TGA) in the 15–220 °C range of Alg-1 material.

Thermogravimetric analyses (TGAs) were also performed for the Alg-1, Alg-2, and Alg-3 products, which exhibited more promising properties. The TGA experiments aim to study the thermal stability of the samples in a larger temperature range (15–220 °C) than the above-mentioned DSC measurements. TGA profile corresponding to Alg-1 is reported in Figure 5, while the analogous results for Alg-2 and Alg-3 are reported in Figures S14 and S15.

The TGA diagram shows an initial moderate mass loss (about 10%) up to 100 °C, which can be attributed to the evaporation of residual moisture, still present in the material despite the dehydration cycle (at 40 °C) carried out prior to analysis. A second, more significant mass loss occurs at T > 150 °C, corresponding to the degradation of alginate functional groups and backbone, as well as of the gelling agents.

A similar TGA profile was obtained for Alg-2, while the weight/temperature plot obtained from the TGA of Alg-3 displays a moderate weight loss (about 10%) up to 150 °C, when the stronger decomposition step takes place.

**Figure 6:**
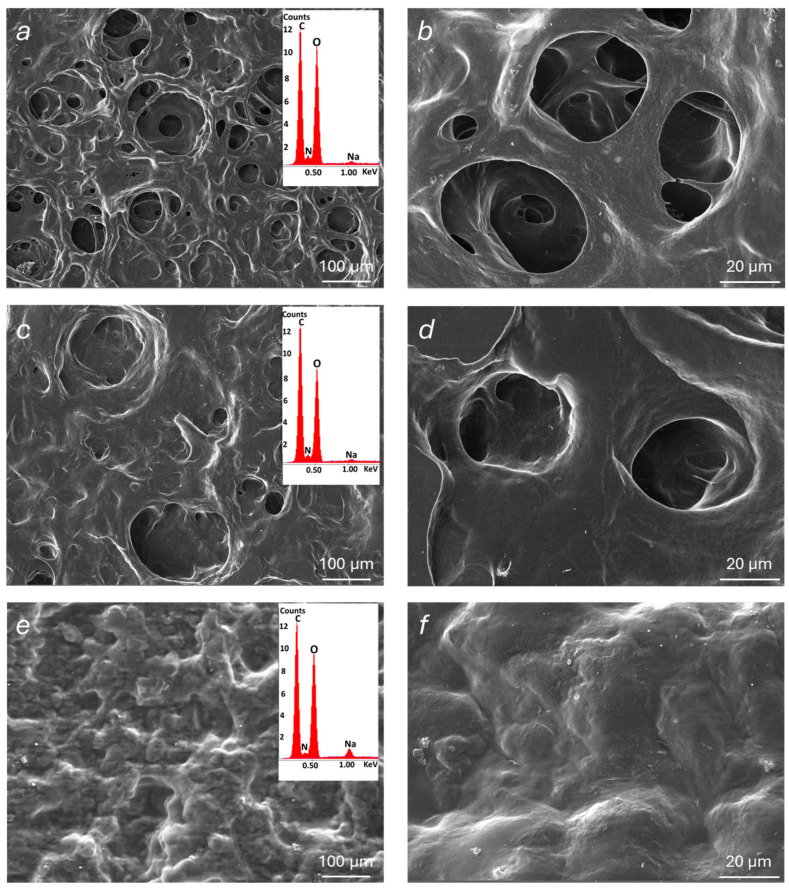
SEM micrographs of the modified alginate materials taken at different magnifications: Alg-1 (**a**,**b**); Alg-2 (**c**,**d**); Alg-3 (**e**,**f**). EDS spectra in the insets.

The morphological and microstructural features of the modified alginate materials were examined by SEM analysis performed on dehydrated samples of polymer films (at 25 °C under vacuum for 16 h). SEM images of Alg-1, Alg-2, and Alg-3, acquired at different magnifications, are reported in Figure 6. Additional images of Alg-4, Alg-6, and Alg-Ca (for comparison purposes) are shown in Figure S16. Alg-5 was not examined by SEM as no stable films can be prepared from this material, which has a powder appearance when dehydrated. Compared to plain Alg-Ca, Alg-1 and Alg-2 exhibit a more porous microstructure, with pores even exceeding 100 µm. In contrast, the surface of Alg-3 shows a more compact morphology, although some traces of pores likely previously open on the surface are still visible. It can be hypothesized that a collapse of Alg-3 porous microstructure occurred during the sample exposition to vacuum. This is consistent with the significantly lower hardness of the Alg-3 hydrogel (see Section 4.3) compared to the other two modified alginate materials. Similarly, SEM images of Alg-4 and Alg-6 show a poorly porous surface, although, in these cases, the absence of pores could be attributed to a possible collapse during the film dehydration process and/or to the high-vacuum measurement conditions. SEM observations in low-vacuum conditions were also attempted, but they did not yield appreciable results due to insufficient resolution.

EDS analyses were also carried out to investigate the elemental composition of the examined materials (insets in Figure 6). The corresponding spectra revealed major peaks associated with carbon and oxygen, along with weaker signals corresponding to nitrogen and sodium. The nitrogen peak further confirms the presence of functional groups resulting from the presence of protonated amines and possibly of NHS. Sodium ions act as the natural counterions of carboxylate groups that do not interact with ammonium species. Moreover, the absence of calcium ions is noteworthy, confirming that crosslinking was mainly mediated by amine-derived ions.

### 3.3. Shape Control of Hydrogel Samples

Gels were prepared from aqueous solutions of the modified alginate as described in Section 2.3. Depending on the molds used during hydrogel preparation, samples can be obtained as films or roughly cubic blocks. Gel samples of Alg-1 and Alg-2 can be prepared with good shape control (film or cube) and retain their shape even after dehydration/rehydration cycles. Alg-3 gel provides both films and bulk samples, although with a less satisfactory shape control. In contrast, Alg-4 showed a limited ability to form films and displayed no shape retention in the case of cubic samples. Alg-6 gel exhibited poor shape definition when attempting to prepare cubic samples and did not provide satisfactory films. Finally, Alg-5 did not display any shape control, being ineffective in forming either films or other bulk materials. These observations suggest that shape control ability of the investigated gels is affected by some polyamine features, i.e., the overall chain length and the number of amino groups (either primary or secondary). Polyamines with longer chain and/or larger number of amines (e.g., compounds 1–3) seem to be more effective in crosslinking the polymer chains and providing good shape control. On the contrary, the gels formed by “short” diamines 5 and 6 do not exhibit shape control, maybe due to a less efficient crosslinking process.

**Figure 7:**
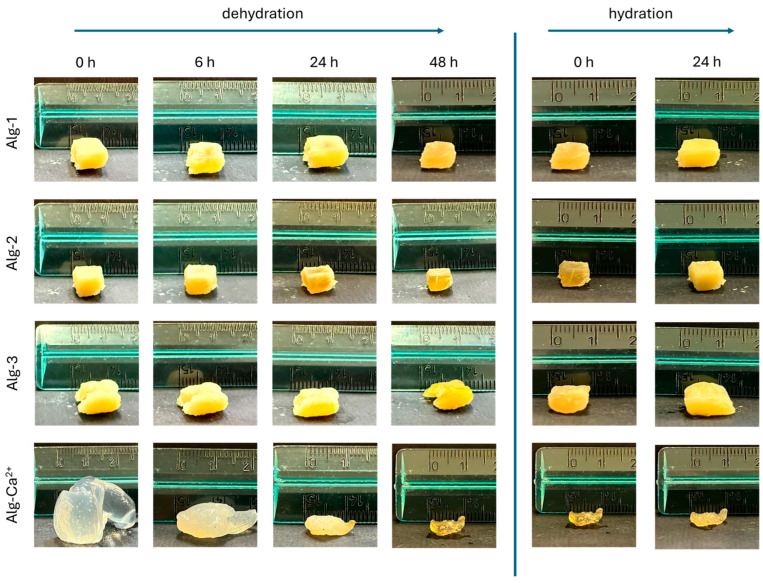
Shape retention of roughly cubic Alg-1, Alg-2 and Alg-3 gel samples during a dehydration/rehydration cycle. The behavior of an Alg-Ca sample is also depicted for comparison.

To demonstrate shape retention during the hydration and dehydration process, Alg-1, Alg-2, and Alg-3 “cubic” samples were fully dehydrated after preparation and then rehydrated. Their dimensions and appearance were checked at different times, as shown in Figure 7, using a ruler. The alginate polymers crosslinked with PEHA, Tren, and Tet were compared with calcium alginate. Alg-1 and Alg-2 display a good shape control, while the behavior of Alg-3 was less satisfactory. Alg-Ca does not exhibit shape retention at all. It suggests that gels obtained from Alg-1 and Alg-2 can be prepared as bulk samples (cubic or different shapes) that are able to preserve their shape even after dehydration and rehydration cycles. In particular, the gel samples do not collapse on the surface where they could be applied even after prolonged time. Moreover, Alg-1 and Alg-2 hydrogels can undergo up to 10 dehydration–hydration cycles without significantly losing their original shapes, while the Alg-3 sample loses its roughly cubic shape after the fifth cycle (Figure S17).

### 3.4. Moisture and Mechanical Properties of Hydrogels

The EWC% and RC analyses were carried out on gel films, in order to minimize the effect of thickness on the results in the case of cubic samples. Results obtained by using the protocols described in Section 2.4 are shown in Table 2, which also reports the EWC% and RC values of calcium alginate, whose moisture properties are widely documented in the literature [65,68]. Hydrogels formed by Alg-4 and Alg-5 did not allow a reliable determination of both parameters.

Water content at equilibrium of gel Alg-1 is very similar to Alg-Ca, while Alg-2 and Alg-6 show EWC% values greater than the reference gel. On the contrary, Alg-3 displays a lower water content than Alg-Ca. All the determined RC values are distinctly larger than calcium alginate, particularly for Alg-1 and Alg-6.

Although the behavior of the investigated materials cannot be easily rationalized as far as their moisture properties are concerned, these results suggest that the capacity of alginate-based hydrogels to adsorb and release water can be tuned depending on the polyamine used as the crosslinker.

Mechanical tests were carried out to monitor the effect of different crosslinking agents on the features of the investigated gels. Tensile tests were performed by using the literature method [69] only on gel films prepared using Alg-1, Alg-2, and Alg-3, because gels obtained from the other investigated materials did not provide suitable films, as mentioned before. However, the measurements were poorly reproducible and the experimental values of tensile strength and elongation at break were characterized by very large errors. It could be due to residual inhomogeneity of the film samples mainly in terms of thickness. Therefore, these measurements were considered not reliable.

**Figure 8:**
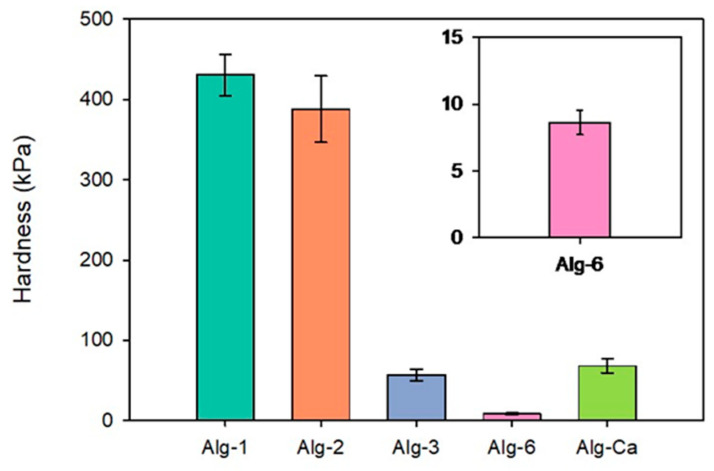
Hardness values (kPa) obtained from compression tests on bulk samples of the gels: Alg-1, Alg-2, Alg-3, Alg-6, and Alg-Ca.

Alg-1, Alg-2, Alg-3, and Alg-6 were also analyzed by compression tests on roughly cubic specimens (1 × 1 × 1 cm^3^). Alg-4 and Alg-5 were not analyzed since their gels cannot be prepared as bulk samples. Results are graphically resumed in Figure 8, where the result obtained on a Alg-Ca sample is reported for comparison. Alg-1 and Alg-2 gels exhibited the highest hardness (around 400 kPa), whereas Alg-3 and Alg-6 showed considerably lower values (about 50 and <10 Kpa, respectively). Moreover, Alg-1 and Alg-2 are distinctly harder than Alg-Ca. These results are consistent with the empirical observations on shape retention reported in Section 4.3 and suggest that HEPA and Tren are more efficient crosslinkers than the other considered polyamines (and then calcium ion).

Viscoelastic properties of Alg-1 and Alg-2 were also investigated to have additional information about the hydrogel network strength. Alg-Ca was also examined, for comparison. Amplitude sweep tests identified the LVR and yield point for all considered hydrogels (Table 3). The Alg-Ca reference gel exhibited an LVR of ~200 Pa and a yield stress of 5.7 kPa. In contrast, the polyamide-crosslinked hydrogels showed significantly higher LVR values (~315 Pa) and a markedly increased yield stress (~75 kPa), indicating a more resistant network and improved mechanical stability. Similar increases in elastic modulus have been correlated with enhanced crosslink density in alginate hydrogels [70].

Frequency-sweep measurements performed within the LVR further confirmed hydrogel formation, with E′ > E″ by approximately one order of magnitude across the tested frequency range, consistent with a predominantly elastic response. Loss factor values (tan δ ≈ 0.23–0.36) remained well below 1 for all formulations, confirming solid-like viscoelastic behavior (Figure S18a). Notably, the polyamine–alginate hydrogels exhibited lower tanδ values than the Alg-Ca control, suggesting a more elastic-dominated and structurally cohesive network resulting from enhanced crosslinking efficiency. Moreover, the extensional storage modulus (E′) and extensional loss modulus (E″) of the gels Alg-1 and Alg-2 and the reference Alg-Ca are reported in Figure S18b–e.

### 3.5. Antifungal Properties

Preliminary laboratory tests pointed out that the modified alginate-based polymers exhibit a clear advantage in terms of resistance to fungal contamination. While calcium alginate, in accordance with what has already been reported in the literature, developed visible fungal colonies within a few days and was entirely colonized after two weeks [44,45], the modified systems Alg-1 and Alg-2 did not show any sign of microbial growth within the same timeframe. In addition, the monitoring period was extended to one month, during which the samples continued to resist fungal attack.

The fungal growth tests were carried out by exposing the samples in open containers to allow natural mold development. The environment was kept humid by spraying distilled water every 24 h to prevent the samples from drying out, and the temperature was maintained at 22 °C. The monitoring was conducted visually and microscopically (OM and SEM) before and after fungal growth.

**Figure 9:**
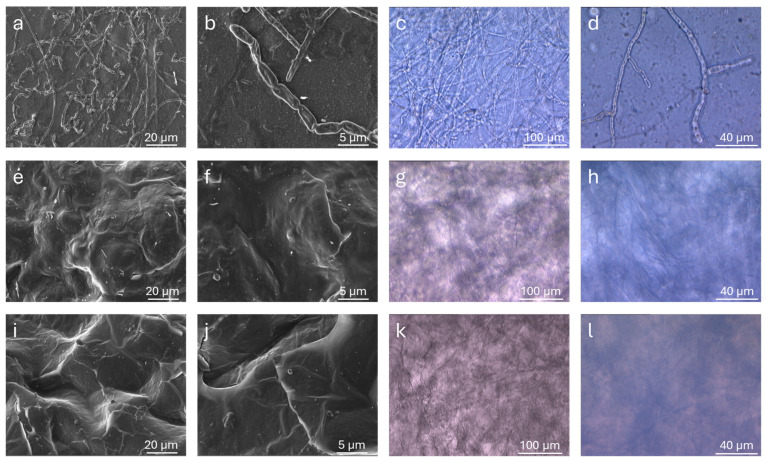
SEM and OM images of Alg-Ca, Alg-1, and Alg-2 hydrogel samples, with different magnification: (**a**,**b**) Alg-Ca, SEM; (**c**,**d**) Alg-Ca, OM; (**e**,**f**) Alg-1, SEM; (**g**,**h**) Alg-1, OM; (**i**,**j**) Alg-2, SEM; (**k**,**l**) Alg-2, OM.

Under an optical microscope, calcium alginate (Alg-Ca) showed the formation of numerous fungal structures after 15 days of the exposition, as shown in Figure 9. Linear, elongated hyphal formations with branched terminations were clearly visible, indicating extensive surface colonization. In contrast, Alg-1 and Alg-2, treated under the same conditions, did not display any fungal colony or surface alterations, confirming their resistance to microbial proliferation. Observations by SEM confirmed the presence of fungal organisms on the surface of Alg-Ca and their absence on the other examined hydrogels. The only noticeable change observed on the Alg-1 and Alg-2 samples was the disappearance of their initial surface porosity, suggesting a partial smoothing or collapse of the pore structure, but with no signs of microbial colonization. The sample was analyzed at different magnifications to confirm the surface coverage, and additional images supporting these observations are reported in Figure S19 of the Supporting Information.

This improved bio-resistance is highly relevant for practical use, particularly when long-term material stability is required. Unlike unmodified calcium alginate, which under humid conditions is vulnerable to colonization, the investigated hydrogels remain intact, allowing for safer handling, easier storage, and lower risk of deterioration in biologically active environments.

Enhanced antimicrobial behavior can be attributed to the presence of ammonium ions within the polymer structure, which likely interfere with fungal adhesion and growth. The stability under high humidity, combined with their notable capacity to absorb and retain water, positions these materials as promising alternatives in fields where sterility and durability are essential. In summary, the resistance to microbial degradation observed in these modified alginate formulations should not be considered merely a protective property: it constitutes a genuine functional improvement that widens their potential use across diverse technological and scientific domains.

## 4. Preliminary Application Experiments

The conservation of historical and artistic artifacts requires targeted cleaning interventions aimed at removing undesired surface deposits without altering the intrinsic properties of the constituent materials. In the case of sensitive substrates such as paper (possibly with ink writings) or wood, even small amounts of dirt or organic residues can compromise the legibility, chemical stability, and physical integrity of the artifact. Controlled methodologies, based on the use of gels and selected solvent systems, allow modulation of the cleaning action and the achievement of reproducible results, while ensuring compatibility with the substrate. Investigations of novel gel materials, along with the definition of standardized protocols of their application, are therefore essential to evaluate the effectiveness of treatments and to develop safe and sustainable approaches for the conservation of cultural heritage. Therefore, the effectiveness of the investigated gel materials as cleaning tools was preliminarily tested on laboratory mock-ups made of artificially soiled shellac-coated wood and iron gall ink-stained paper.

### 4.1. Preparation of Mock-Ups and Evaluation of Preliminary Results

The preparation of mock-ups represents an essential preliminary step for the evaluation of cleaning methodologies on substrates of historical and artistic interest. In this study, paper samples stained by iron gall ink and shellac-finished wood specimens were specifically prepared as representative of two kinds of very sensitive cultural heritage items, i.e., paper manuscripts and wood artifacts. The surface of the mock-ups was treated with artificial dust as a simulant of soiling deposits. The use of such a simulant, widely documented in the literature, allows the standardization of experimental protocols and the controlled reproduction of soiling conditions typically found on genuine artifacts [71].

Alg-1 and Alg-2 gels were selected to carry out the preliminary test for the removal of the soil applied on the laboratory mock-ups. In fact, films obtained from these materials are easily applicable on the surface of different substrates (e.g., wood, paper, and stone) and can be easily removed without breaking even after prolonged exposition (e.g., up to 12 h) at ambient conditions. Moreover, as shown by visual and microscope observation, no gel residues remain on the surface after film removal.

Following the controlled application of the hydrated gels Alg-1 and Alg-2 as cleaning tools (Section 2.6) on mock-ups soiled by artificial dust, the preliminary evaluation focused on the effectiveness of the cleaning and the compatibility of the treatment with the substrates. The treated surfaces were characterized before and after cleaning by means of colorimetry and μ-FT-IR spectroscopy [62,63,72] in order to quantify chromatic variations resulting from the treatment and to detect possible residual components on the surface after the cleaning process.

Visual observation and optical microscope (OM) monitoring were also performed to obtain further information on the effect of the cleaning procedure (e.g., uniformity of the soil removal). These preliminary tests enabled us to evaluate the performance of the cleaning tools obtained by the combination of Alg-1 and Alg-2 gels with the considered solvent systems (i.e., 1-butanol/water 1:9 *v*/*v*, ethanol/water 1:9 *v*/*v*, 1% Ecosurf^®^ in water, nanoemulsion composed of 9.7% 1-butanol, 20.9% butanone, and 3.5% Ecosurf^®^ in water [60], and demineralized water).

### 4.2. Cleaning of Shellac-Finished Wood

Maple specimens varnished by shellac were examined at three different stages: (i) before soiling; (ii) after application of artificial dust; and (iii) after the cleaning process by the selected gel/solvent systems performed as described in Section 2.5. This approach allowed for the assessment of both the impact of the dust on the varnished wooden surface and the effectiveness of the gels in removing deposits without altering the chemical or chromatic properties of the underlying substrate. An image depicting a wood specimen soiled by artificial dust and after application of different gel/solvent cleaning systems is reported, as an example, in Figure S20a,b.

Results obtained by colorimetric analyses are graphically represented in Figure S21, in which ΔE* values refer to the overall chromatic variations obtained by comparing the cleaned and not-soiled surfaces. The colorimetric coordinates (L*, a*, b*) measured on wood samples coated with shellac, on shellac-coated wood after soiling with artificial dust deposition, and after cleaning with different solvents loaded in the Alg-1, Alg-2, and Alg-Ca hydrogels are reported in Table S1–S3, respectively. Table S4 reports the ΔE* values obtained after the cleaning tests performed with Alg-1, Alg-2, and Alg-Ca gels using the different solvent mixtures. As a first approximation, we can assume that the lower the ΔE* value is, the more efficient the cleaning tool can be considered in removing the soil from the surface. When Alg-1 is considered, the combination with Ecosurf^®^/water system seems to be the most effective since the chromatic variation observed after the cleaning corresponds to about ΔE* = 5 [64]. In fact, when Alg-1 hydrogel was loaded with other solvent mixtures, significantly higher ΔE* values were observed. In particular, demineralized water proved to be the least effective solvent in removing artificial dust. The effectiveness of Alg-1 loaded with Ecosurf^®^/water is also confirmed by OM observation: after application of the cleaning system, the examined surface is free from the dark particles of the artificial dust (Figure S20c,d).

All the cleaning systems based on Alg-2 induced overall chromatic variations larger than 8 after the cleaning application, suggesting a not completely satisfactory soil removal.

The combination of Alg-1 with 1% Ecosurf^®^ in water was considered the most promising cleaning system for the controlled cleaning of wood specimens and it was further investigated by μ-FT-IR spectroscopy.

**Figure 10:**
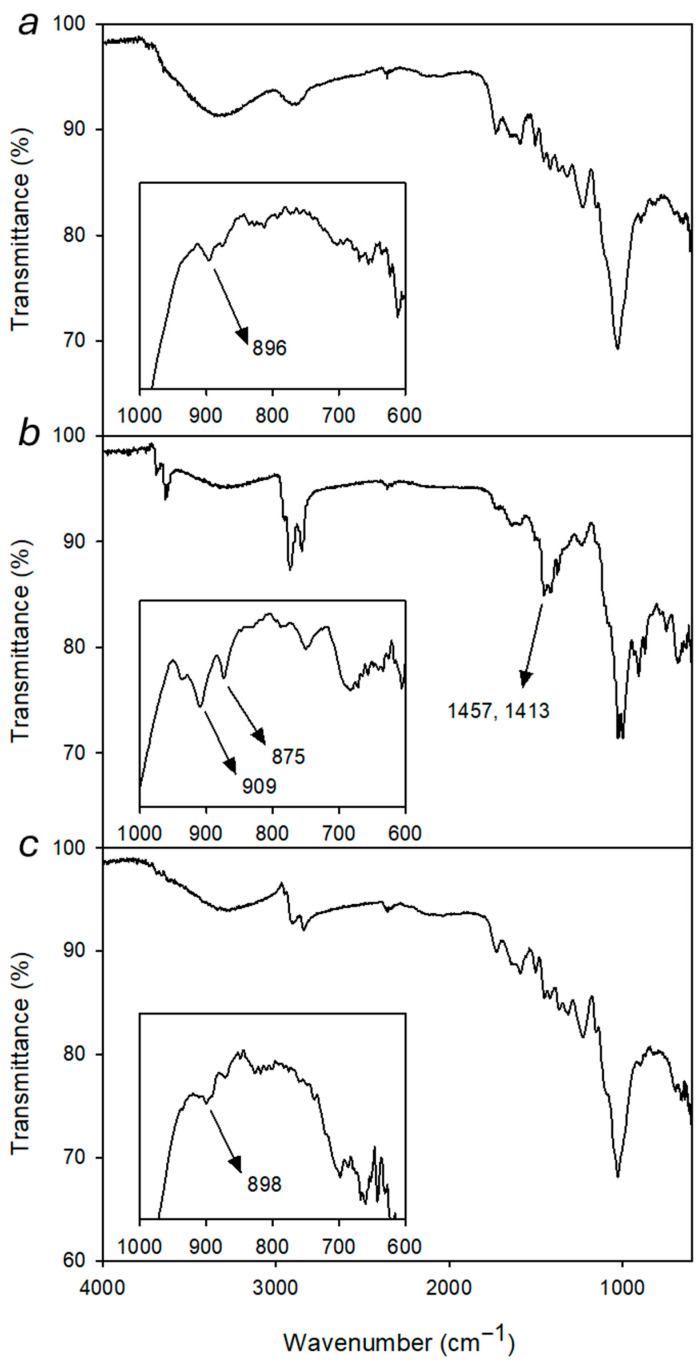
μ-FT-IR spectra of (**a**) shellac-coated wood, (**b**) shellac-coated wood treated with artificial dust, (**c**) shellac-coated wood after the cleaning procedure by Alg-1 with 1% Ecosurf^®^ in water and with magnified views of the spectral region between 1000 and 600 cm^−1^.

μ-FT-IR spectrum of shellac-coated maple surface (Figure 10a) shows characteristic bands attributable to the wooden substrate and to the organic surface coating. The broad band at 3500–3000 cm^−1^ can be ascribed to the stretching of hydroxyl groups (O–H) belonging either to different components (shellac, cellulose, and hemicellulose) and to residual moisture. The peak at 2895 cm^−1^ corresponds to the stretching of aliphatic C–H, while the weak bands between 1727 and 1582 cm^−1^ include the C=O stretching of carbonyl and carboxyl groups (lignin, hemicellulose, and shellac) as well as the C=C aromatic vibrations of lignin. The signal at 1240 cm^−1^ can be attributed to C–O stretching of alcoholic and ester groups, consistent with the presence of the shellac-based coating. Finally, the peaks at 1023 cm^−1^ and 896 cm^−1^ correspond to the C–O–C absorptions in cellulose, typical of wooden materials.

After deposition of artificial dust, significant differences can be observed in the FT-IR spectrum taken on the surface of the wood specimen, when compared with the unsoiled sample (Figure 10b). The peak at 3622 cm^−1^ is assigned to O–H stretching of hydrated silicates, while the broad band centered at about 3300 cm^−1^ still concerns hydroxyl groups from the underlying substrates. Bands in the range 2950–2800 cm^−1^ ascribed to stretching of aliphatic C–H undergo an intensity increase due to the presence of paraffin component in the dust. The absorption at 1460–1400 cm^−1^ can be ascribed to C–H bending of organic components [58] with possible contributions from stretching vibrations of carbonate formed from calcium hydroxide originally included in the artificial dust. The strong band at 1014 cm^−1^ is characteristic of Si–O stretching in silicates and is partially overlapped on the C-O-C band observed in the substrate spectrum. The new peaks that appeared in the 1000–600 cm^−1^ range are mainly due to absorptions of siliceous components. [73] In particular, the peak at 909 cm^−1^ is consistent with Kaolin Al-O-H deformation vibration. Finally, the peak 875 cm^−1^ is correlated to bending vibration in calcite [74].

After cleaning by Alg-1 loaded with 1% Ecosurf^®^ in water, the spectrum again shows the characteristic absorptions of the wooden substrate and shellac, without significant signals from the dust components (Figure 10c). Bands are observed at 3266 cm^−1^ (O–H stretching of cellulose, hemicellulose, and moisture), and at 2856 cm^−1^ (aliphatic C–H stretching), values which are typical of treated wood without the anomalous intensity observed in the dust spectrum. Weak bands between 1718 and 1583 cm^−1^ confirm the presence of carbonyl and aromatic groups from lignin and ester groups from shellac. 

The signal at 1242 cm^−1^ is assigned to C–O stretching of alcohols and esters, while the intense peak at 1024 cm^−1^ corresponds to the C–O–C stretching of cellulose. In particular, the absence of the bands at 1460–1400 cm^−1^, 909 cm^−1^ and 875 cm^−1^, ascribed to the dust components, suggests a satisfactory removal of organic and inorganic compounds of the artificial soil from the surface. Following the cleaning treatment, the peak C-O-C at 898 cm^−1^ of the wooden materials becomes detectable again (Figure 10c inset).

### 4.3. Cleaning of Iron Gall Ink on Paper

Colorimetric and FT-IR analyses were also used to preliminarily evaluate the effectiveness of Alg-1 gel in the removal of artificial dust from paper mock-ups simulating manuscript samples. Considering that iron gall ink is highly soluble in both water and hydroalcoholic solutions, at first the effect of the different solvents on ink was tested. Gel films loaded with the considered solvents were applied on the paper/ink mock-up and the chromatic variation expressed as ΔE* was measured after the gel removal. Ink dissolution and migration into the hydrogel phase was induced by all solvents (with significant ΔE* values) except for ethanol/water 1:9 *v*/*v* mixture, whose application induced only a very low chromatic change (ΔE* < 1). A picture displaying a paper/ink mock-up before and after application of Alg-1 film loaded with the hydroalcoholic solution is shown in Figure S22, where the discoloration of the mock-up after treatment by plain hydrated gel is also depicted for comparison. The effect of the application of this gel on the ink was then investigated by colorimetric and FT-IR analyses. The values of the chromatic variations observed after three consecutive applications of the gel are presented in Figure S23 and Table S5. Given the very small ΔE* values, we assumed that ethanol/water 1:9 *v*/*v* was substantially inactive towards the ink matrix. This was also confirmed by the infrared spectra taken on the ink-paper mock-ups before and after application of the gel loaded with hydroalcoholic solution, which shows no significant differences (Figure S24). Therefore, Alg-1 gel loaded with ethanol/water 1:9 *v*/*v* was selected for further cleaning tests, i.e., for the removal of artificial dust deposited on the iron gall ink mock-ups.

The result of the cleaning obtained after 2 min application of this cleaning tool on the ink-paper mock-ups is presented in Figure S25, where the cleaned area is compared to the native ink-paper surface and to the same surface soiled by artificial dust.

The spectrum of plain Whatman paper is also presented in Figure S26, for comparison. It displays characteristic cellulose peaks, which can also be seen in the mock-up spectrum: 3330–3400 cm^−1^ (O–H stretching and moisture), 2900 cm^−1^ (C–H stretching), 1050–1100 cm^−1^ (C–O–C stretching), 1200–1250 cm^−1^ (alcoholic C–O stretching), and 800–900 cm^−1^ (aromatic C–H deformation, associated with residual lignin traces in the paper).

The effectiveness of the cleaning process was further investigated by μ-FT-IR technique. The spectra taken on original ink-paper mock-ups are reported in Figure S27a. Absorptions in the 3330–3400 cm^−1^ region are due to O–H stretching of cellulose and residual moisture. The carbonyl groups of tannins are manifested by a peak between 1700 and 1720 cm^−1^, while the 1610–1640 cm^−1^ band is assigned to aromatic C=C stretching of phenolic compounds. The formation of iron–tannate complexes is evidenced by the band at 1510–1530 cm^−1^, together with the 1300–1350 cm^−1^ peak, representative of C–O stretching of phenolic groups coordinated with Fe^3+^. Other relevant peaks include 1200–1250 cm^−1^ (phenolic C–O stretching), 1050–1100 cm^−1^ (C–O–C stretching of cellulose), 1380–1400 cm^−1^ (Fe^3+^–O–C interactions), and finally 800–900 cm^−1^, corresponding to aromatic C–H deformation.

After soiling, μ-FT-IR spectrum (Figure S27b) displays the contribution of artificial dust to the overall absorption. The intense peak at about 1000 cm^−1^ is attributed to Si–O stretching of silicates in the artificial dust, while the 3337 cm^−1^ band reflects O–H stretching of silica, confirming the presence of hydrated inorganic components. The peaks at 2950–2850 cm^−1^ correspond to aliphatic C–H stretching of the organic dust components (e.g., paraffin). The characteristic peaks of iron gall ink, although of low intensity, can be still observed at 1692 cm^−1^ (C=O stretching of tannins), 1602 cm^−1^ (aromatic C=C of tannins), 1448 cm^−1^ (aromatic C–H deformation and Fe^3+^–tannate bonds), 1371 cm^−1^ (Fe^3+^–O–C interactions), and 1307 cm^−1^ (phenolic C–O stretching coordinated with Fe^3+^), along with the peak at 754 cm^−1^ assigned to aromatic C–H deformation. The relatively low intensity of these bands indicates that the ink is present but partially masked by the artificial dust.

Following cleaning performed with the gel (Figure S27c), the spectrum again shows the characteristic peaks of cellulose and iron gall ink described above, while the silica-related signals from the artificial dust (particularly the intense Si–O stretching at about 1000 cm^−1^ and the O–H stretching at 3337 cm^−1^) completely disappear. This confirms the effective removal of the deposits and the preservation of the chemical features of both the ink and the paper.

## 5. Conclusions

The conservation of historical and artistic artifacts requires targeted cleaning interventions aimed at removing undesired surface deposits without altering the intrinsic properties of the constituent materials. In the case of sensitive substrates such as paper (possibly with ink writings) or wood, even small amounts of dirt or organic residues can compromise the legibility, chemical stability, and physical integrity of the artifact. For instance, even simple dust, accumulated on the surface due to poor storage conditions, when combined with ambient humidity, may form patinas that are often aggressive and difficult to remove.

Controlled methodologies, based on the use of gels and selected solvent systems, allow the modulation of the cleaning action and achieve reproducible results, while ensuring compatibility with the substrate. Investigation on novel gel materials along with the definition of standardized protocols of their application are therefore essential to evaluate the effectiveness of treatments and to develop safe and sustainable approaches for the conservation of cultural heritage. For this purpose, novel hydrogels have been prepared from natural polymer alginate, which has been physically crosslinked with aliphatic polyamines in the presence of N-hydroxysuccinimide. The alginate-based materials display distinctly higher transition temperature (T*_g_*) than plain alginate, and some of them show a very large porosity at a microscopic level, supporting the effectiveness of the crosslinking process that modifies the features of the polymer network. The novel hydrogels exhibit different moisture and mechanical properties that can be tuned depending on the selected polyamine. In addition, some of them can be prepared as bulk samples (e.g., film or roughly cubic), which display a good shape retention even after repeated dehydration/hydration cycles (up to 10 cycles). Differently from the plain alginate, all the investigated hydrogels also display resistance to the attack of microbiological agents such as common mold, which can be due to the presence of polyammonium compounds encompassed in the polymeric matrix. Preliminary tests performed on the laboratory mock-ups showed that at least one of the investigated gels displays promising features to be applied in the preparation of cleaning tools for the conservation of sensitive cultural heritage items. In fact, Alg-1 loaded with 1% Ecosurf^®^ in water and with ethanol/water 1:9 *v*/*v* was effective in removing soil from the surface of shellac-coated wood and from ink-stained paper, respectively.

In conclusion, the introduction of polyammonium species into the alginate matrix allows the formation of hydrogel materials with good shape control, providing, at the same time, protection from microbial degradation and good cleaning effectiveness particularly when sensitive surfaces are considered.

Further experiments are currently being carried out to test the applicability of the investigated hydrogels as cleaning systems in real-world cases within the cultural heritage field.

## Figures and Tables

**Table 1:** Results of DSC analyses of the alginate-based materials investigated.

Polymers	Transition Temperatures (T_g_, °C)
Alg-1	56.2
Alg-2	57.2
Alg-3	56.8
Alg-4	54.9
Alg-5	54.5
Alg-6	55.8

**Table 2:** EWC% and RC values determined for the investigated alginate hydrogels.

Polymers	EWC%	RC (mg/cm^2^)
Alg-1	66.14 ± 0.04	24.2 ± 2.1
Alg-2	76 ± 2	14.7 ± 0.7
Alg-3	43 ± 3	15.8 ± 0.5
Alg-4	n.a. ^a^	n.a. ^a^
Alg-5	n.a. ^a^	n.a. ^a^
Alg-6	90 ± 1	21 ± 1
Alg-Ca	65.0 ± 5.0	8.4 ±1.0

^a^ not acquired.

**Table 3:** LVR and yield point of the hydrogels.

Samples	LVR (Pa)	Yield Point (kPa)
Alg-Ca	205	5.7 ± 0.1
Alg-1	315	75.4 ± 0.2
Alg-2	310	74.8 ± 0.1

## Data Availability

The data presented in this research study are available in the present article and in the related Supplementary Information.

## Supplementary Materials

The following supporting information can be downloaded at: https://www.mdpi.com/article/10.3390/polym17222976/s1, Figure S1: FT-IR spectrum (ATR mode) of Alg-Na; Figure S2: FT-IR spectrum (ATR mode) of Alg-1; Figure S3; FT-IR spectrum (ATR mode) of Alg-2; Figure S4 FT-IR spectrum (ATR mode) of Alg-3; Figure S5: FT-IR spectrum (ATR mode) of Alg-4; Figure S6: FT-IR spectrum (ATR mode) of Alg-5; Figure S7: FT-IR spectrum (ATR mode) of Alg-6; Figure S8: DSC analysis of Alg-1 performed in the 15–65 °C range; Figure S9: DSC analysis of Alg-2 performed in the 15–65 °C range; Figure S10: DSC analysis of Alg-3 performed in the 15–65 °C range; Figure S11: DSC analysis of Alg-4 performed in the 15–65 °C range; Figure S12: DSC analysis of Alg-5 performed in the 15–65 °C range; Figure S13: DSC analysis of Alg-6 performed in the 15–65 °C range; Figure S14: TGA of Alg-2 performed between 15 and 220 °C; Figure S15: TGA of Alg-3 performed between 15 and 220 °C; Figure S16: SEM micrographs of Alg-4 (a,b), Alg-6 (c,d), and Alg-Ca (e,f) at different magnifications; Figure S17: Visual appearance of the Alg-1, Alg-2, and Alg-3 polymer samples after 1, 3, 5, and 10 hydration–dehydration cycles. The images show the gels following the hydration phase of each cycle. Figure S18: Comparison of the loss factors, respectively, storage modulus and loss modulus for Alg-Ca, Alg-1, and Alg-2 gels; Figure S19: SEM and OM images of Alg-Ca, Alg-1, and Alg-2 hydrogel samples after antimicrobial assessment test; Figure S20: Picture of shellac-coated specimen after deposition of artificial dust and after treatment with Alg-1 gel loaded with selected solvents; Figure S21: Colorimetric analysis of wood samples with artificial dust; Figure S22: Paper/Iron gall ink mock-up before the gel application, after the application of Alg-1 gel loaded with hydro-alcoholic solution, and the application of Alg-1 gel loaded with the water; Table S1: Colorimetric parameters (L*, a*, b*) of wood samples coated with shellac; Table S2: Colorimetric parameters (L*, a*, b*) of wood samples coated with shellac and soiled; Table S3: Colorimetric parameters (L*, a*, b*) measured on the wood surfaces after cleaning with different solvent mixtures; Table S4: ΔE* values measured on wood surfaces after cleaning with different solvents; Figure S23: ΔE* values obtained by the colorimetric analysis of a paper/iron gall ink after consecutive application of Alg-1 gel loaded with ethanol/water 9:1 *v*/*v*; Table S5: Colorimetric parameters (L*, a*, b*) of the ink surface before artificial soil deposition, and after the cleaning; Figure S24: Micro-FT-IR spectra of Iron gall ink before and after the application of the gel Alg-1 loaded with ethanol/water 1:9 *v*/*v*; Figure S25: Mock-ups of iron gall ink and Alg-1 gel after the cleaning process; Figure S26: Micro-FT-IR spectrum of Whatman 1 paper; Figure S27: Micro-FT-IR spectra of iron gall ink on paper, iron gall ink on paper treated with artificial dust, and paper-ink sample after the application of Alg-1 gel loaded with ethanol/water 1:9 *v*/*v*.
