# Supplementary material for: Novel Alginate-Based Physical Hydrogels: Promising Cleaning Tools for Sensitive Artifacts
Source: Polymers (Basel). 2025 Nov 8;17(22):2976. doi: 10.3390/polym17222976 (PMC12655926; doi:10.3390/polym17222976)
Supplement: Supplementary file 1 [file polymers-17-02976-s001.zip › polymers-3932792-supplementary.pdf]

NOVEL ALGINATE-BASED PHYSICAL HYDROGELS: PROMISING CLEANING TOOLS FOR SENSITIVE ARTIFACTS

Matteo Ferretti, Maduka L. Weththimuni,* Donatella Sacchi, Chiara Milanese, Alessandro Girella, Barbara Vigani, Gaia Zucca, Alice Pedalà, Nicola Razza, Maurizio Licchelli*

1. Molecular analysis

1.1 FT-IR analysis FT-IR spectra of Alg-1, Alg-2, Alg-3, Alg-4, Alg-5, Alg-6 and of the reference Alg-Na.

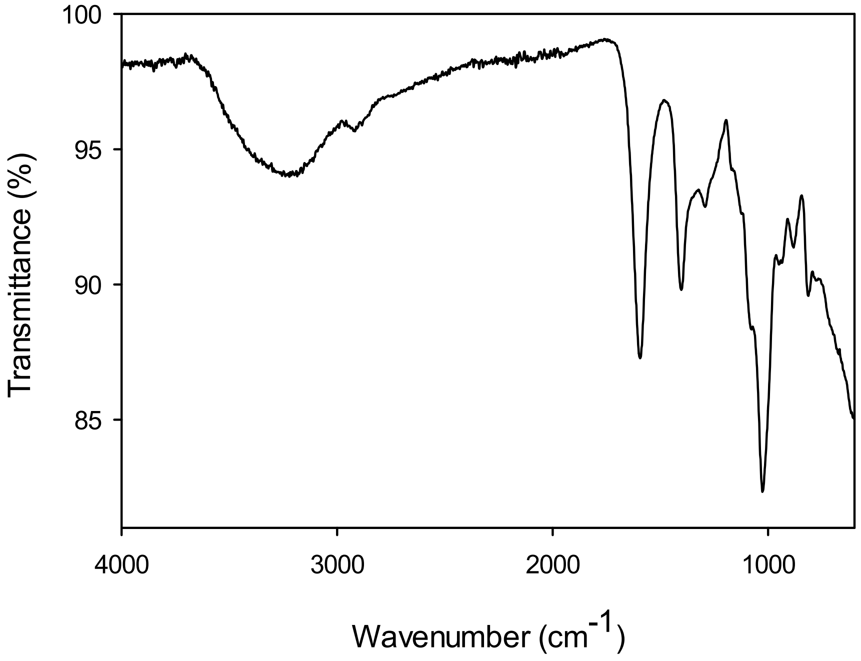

Figure S1. FT-IR spectrum (ATR mode) of Alg-Na.

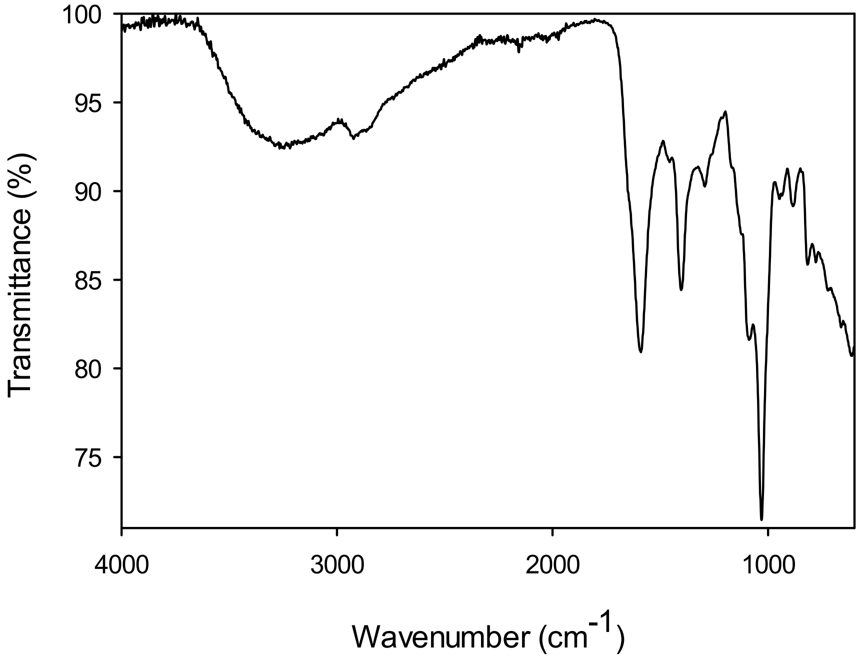

Figure S2. FT-IR spectrum (ATR mode) of Alg-1.

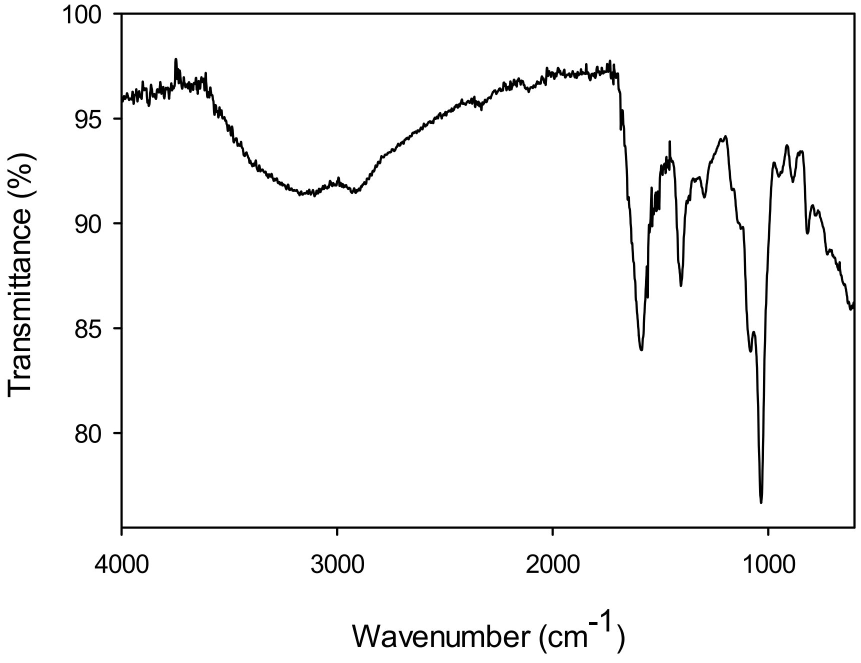

Figure S3. FT-IR spectrum (ATR mode) of Alg-2.

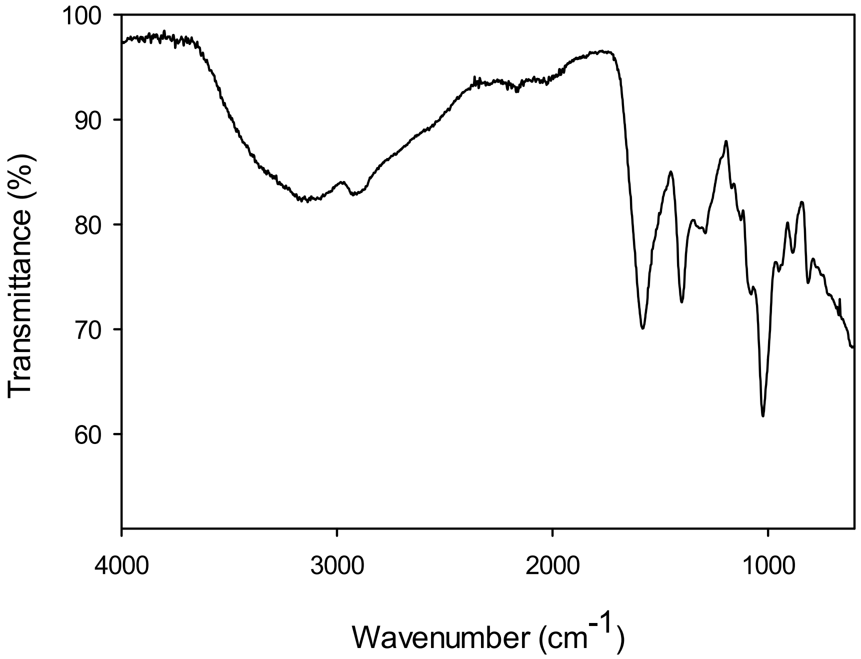

Figure S4. FT-IR spectrum (ATR mode) of Alg-3.

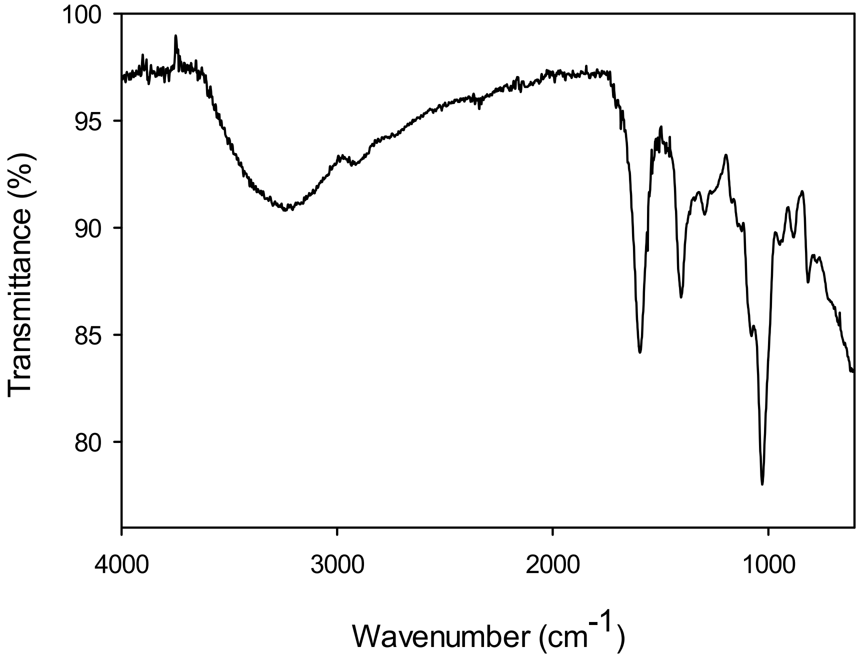

Figure S5. FT-IR spectrum (ATR mode) of Alg-4.

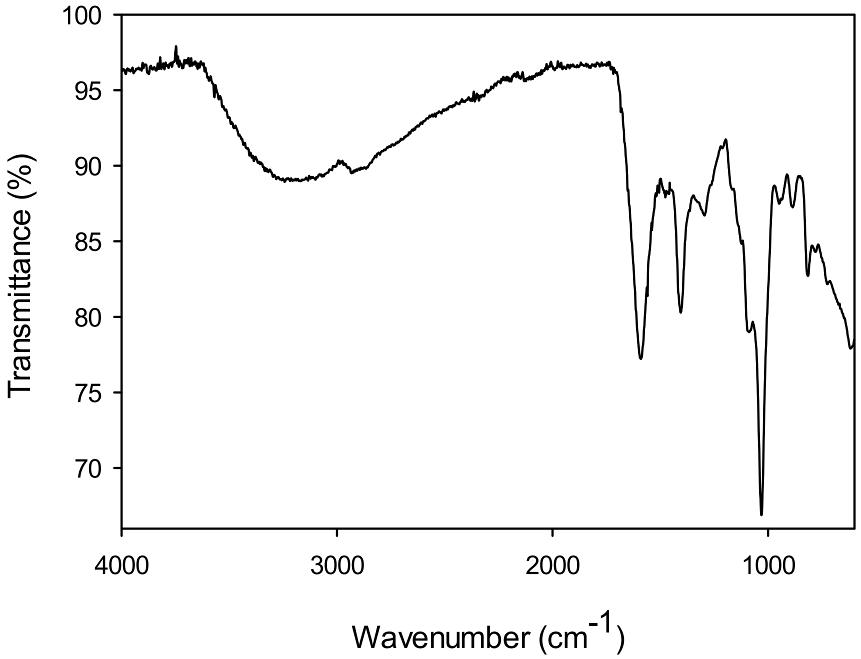

Figure S6. FT-IR spectrum (ATR mode) of Alg-5.

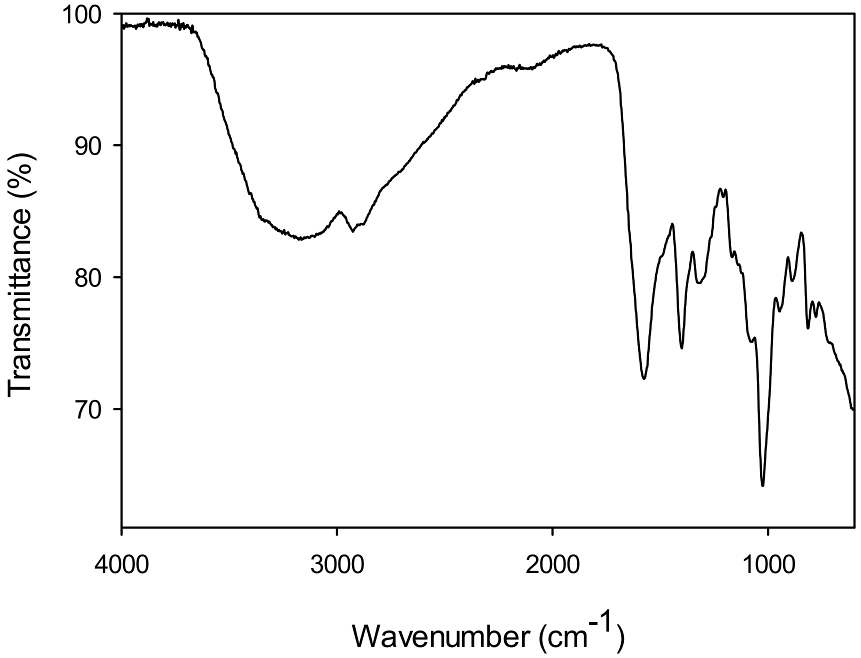

Figure S7. FT-IR spectrum (ATR mode) of Alg-6.

2. Hydrogels characterization

This section reports the characterization analyses of the gels prepared from the modified alginate polymers. Thermal analyses are reported, as well as SEM images and the moisture properties.

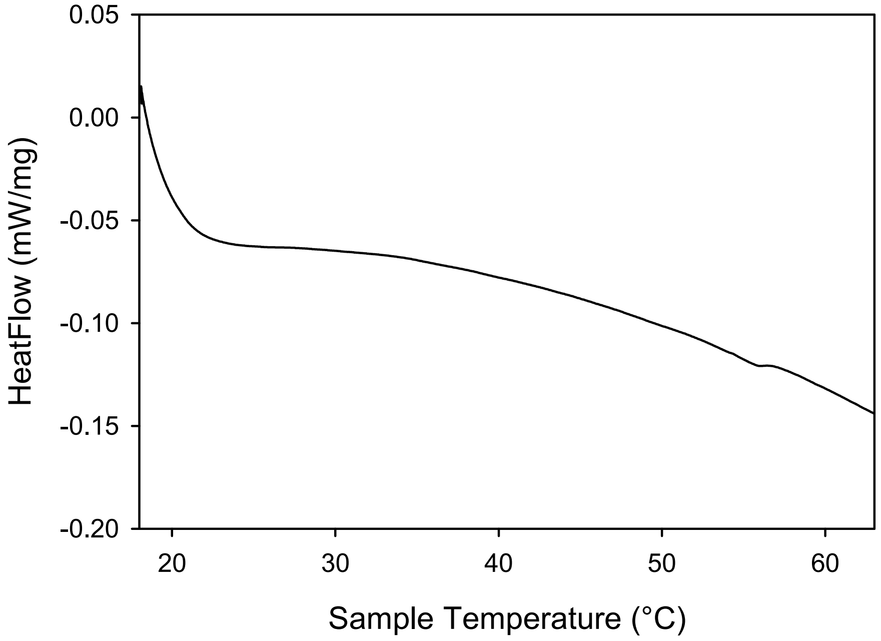

Figure S8. DSC analysis of Alg-1 performed in the 15–65 °C range.

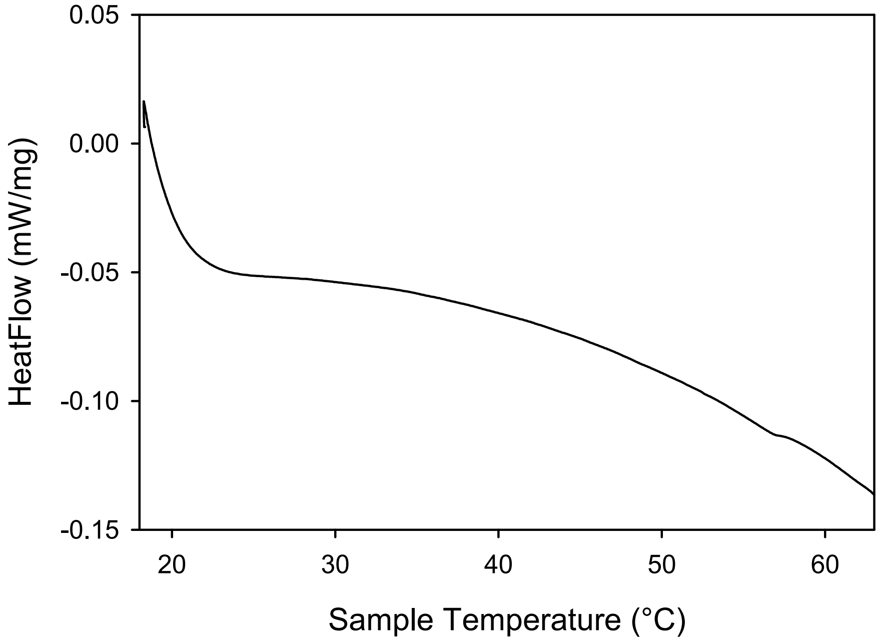

Figure S9. DSC analysis of Alg-2 performed in the 15–65 °C range.

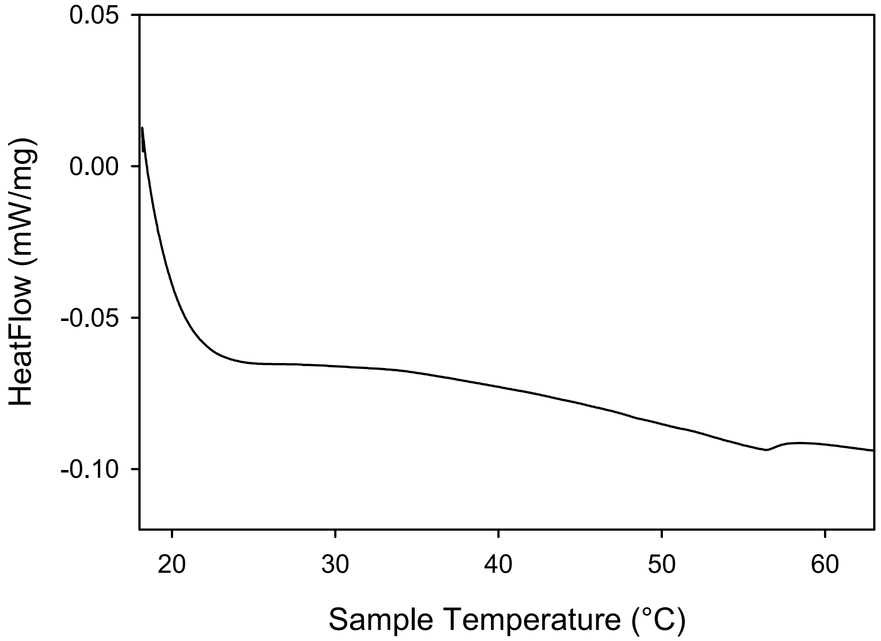

Figure S10. DSC analysis of Alg-3 performed in the 15–65 °C range.

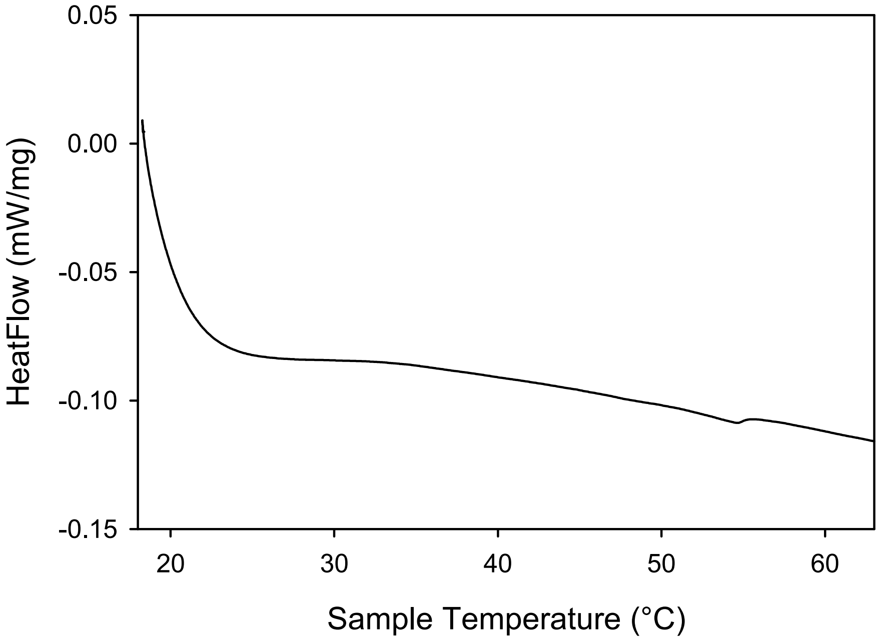

Figure S11. DSC analysis of Alg-4 performed in the 15–65 °C range.

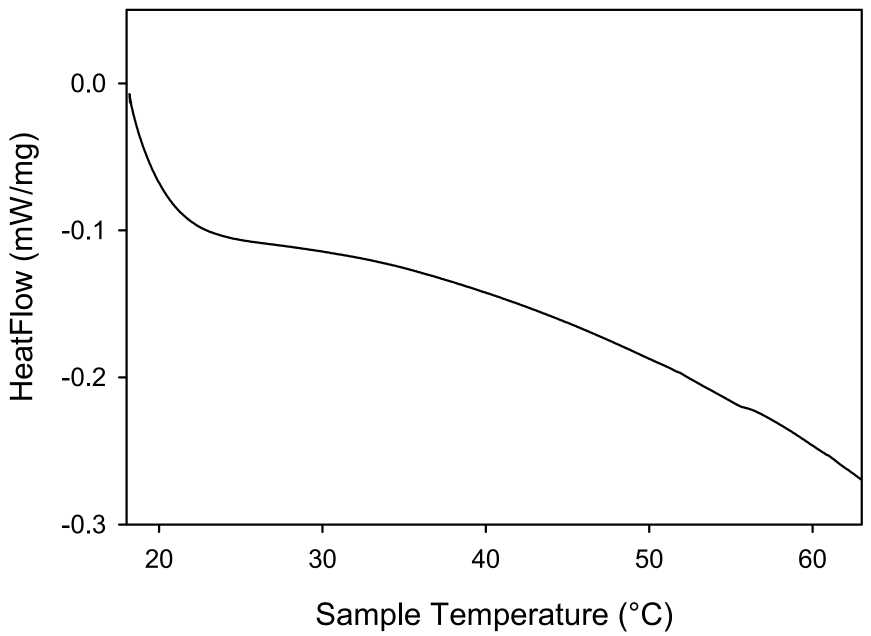

Figure S12. DSC analysis of Alg-5 performed in the 15–65 °C range.

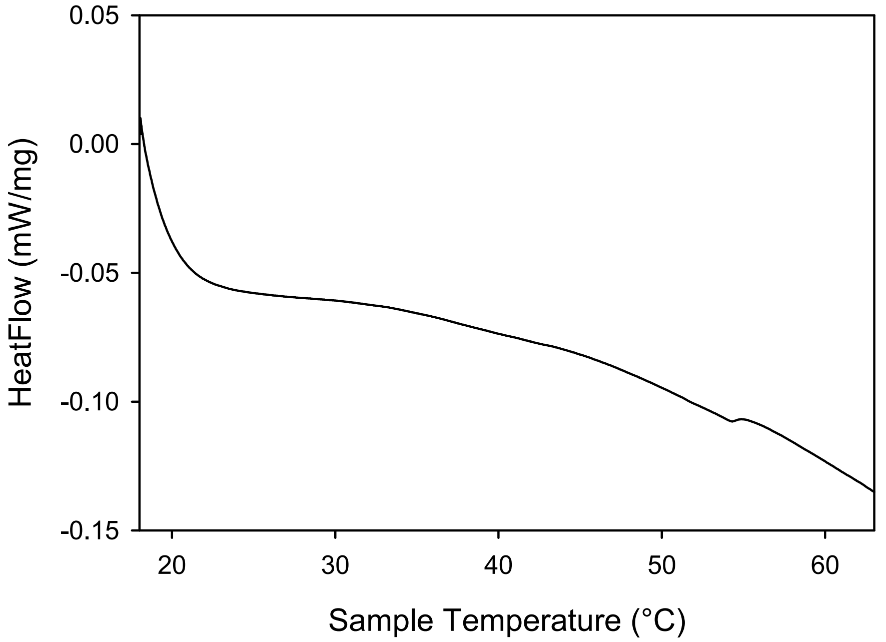

Figure S13. DSC analysis of Alg-6 performed in the 15–65 °C range.

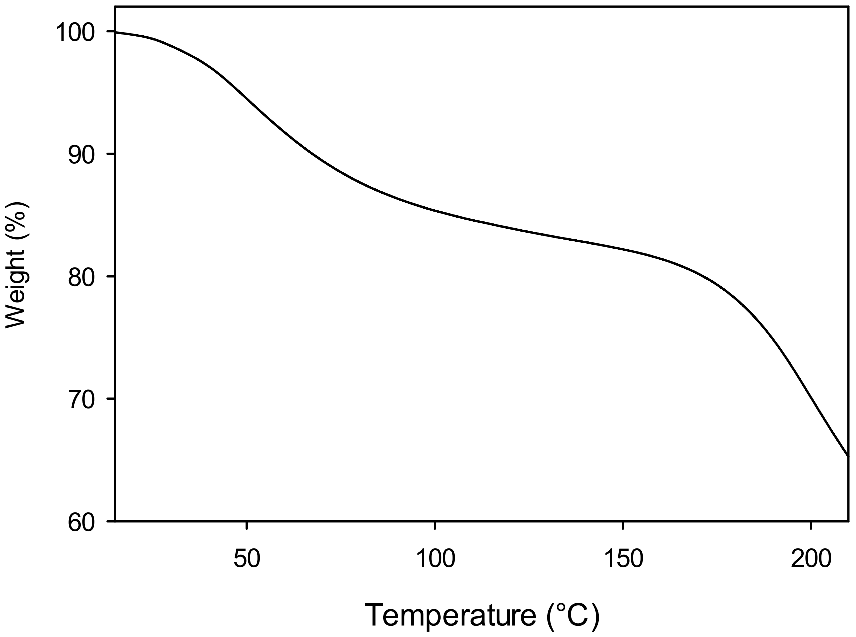

Figure S14. TGA analysis of Alg-2 performed between 15 and 220 °C.

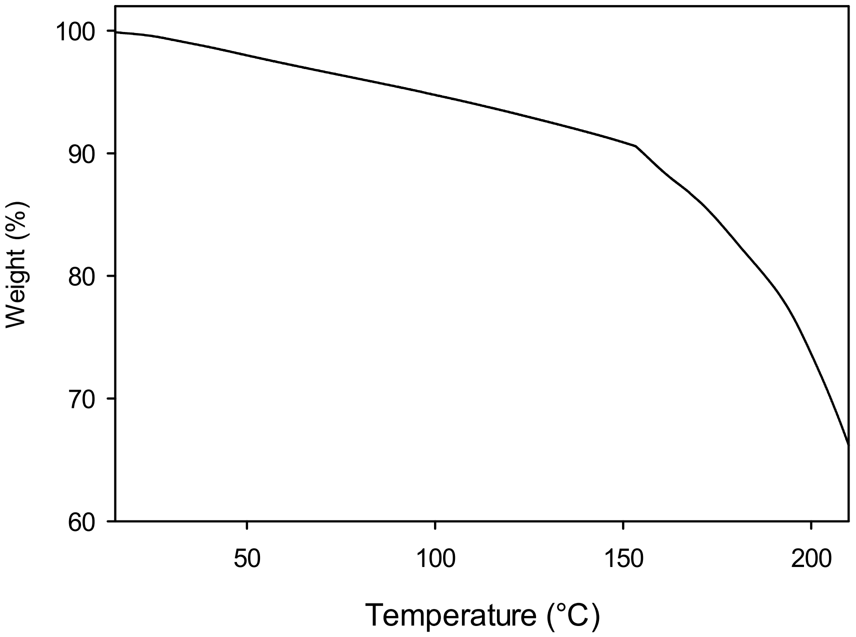

Figure S15. TGA analysis of Alg-3 performed between 15 and 220 °C.

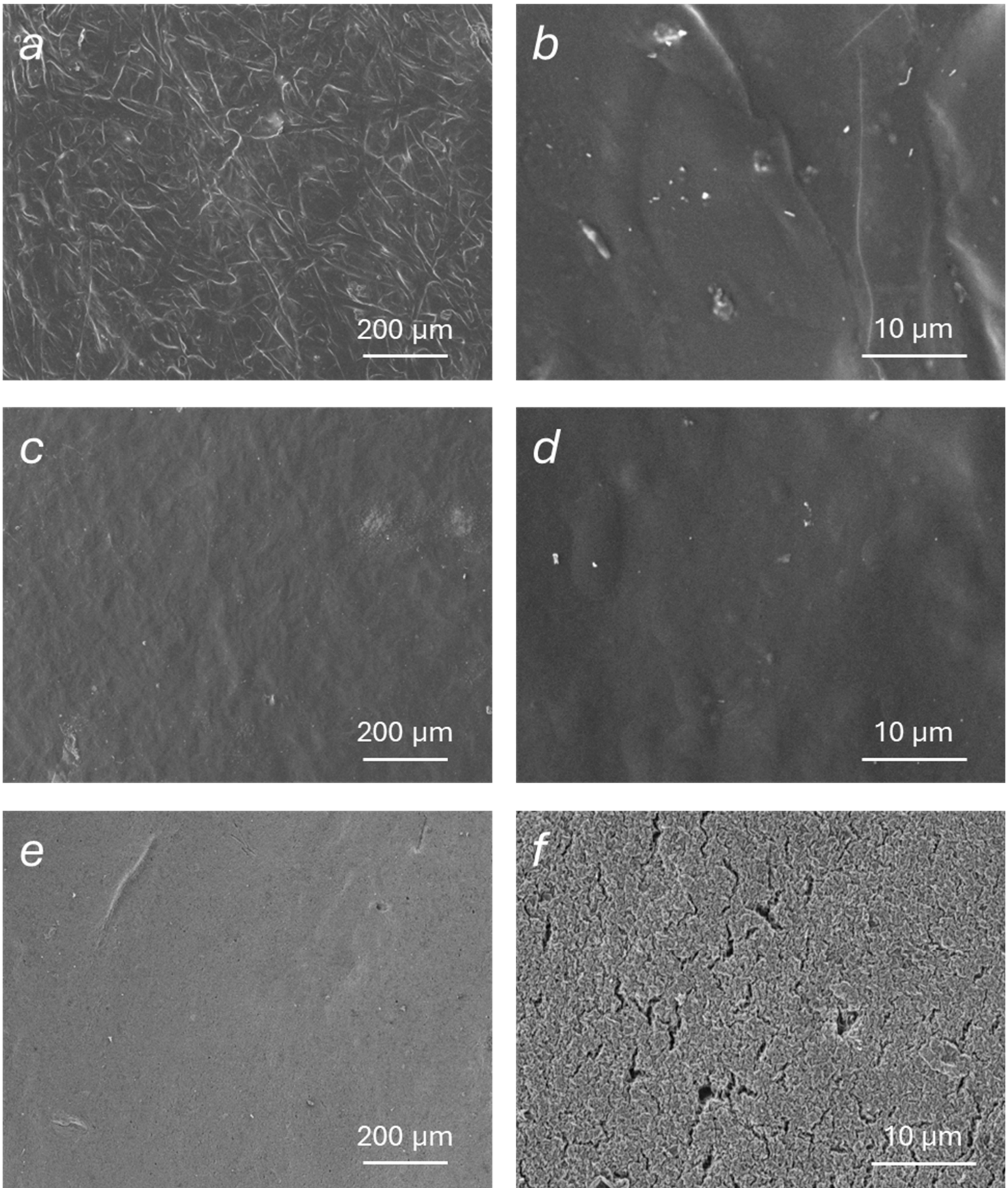

Figure S16. SEM micrographs of Alg-4 (a,b), Alg-6 (c,d), and Alg-Ca (e,f) at different magnifications.

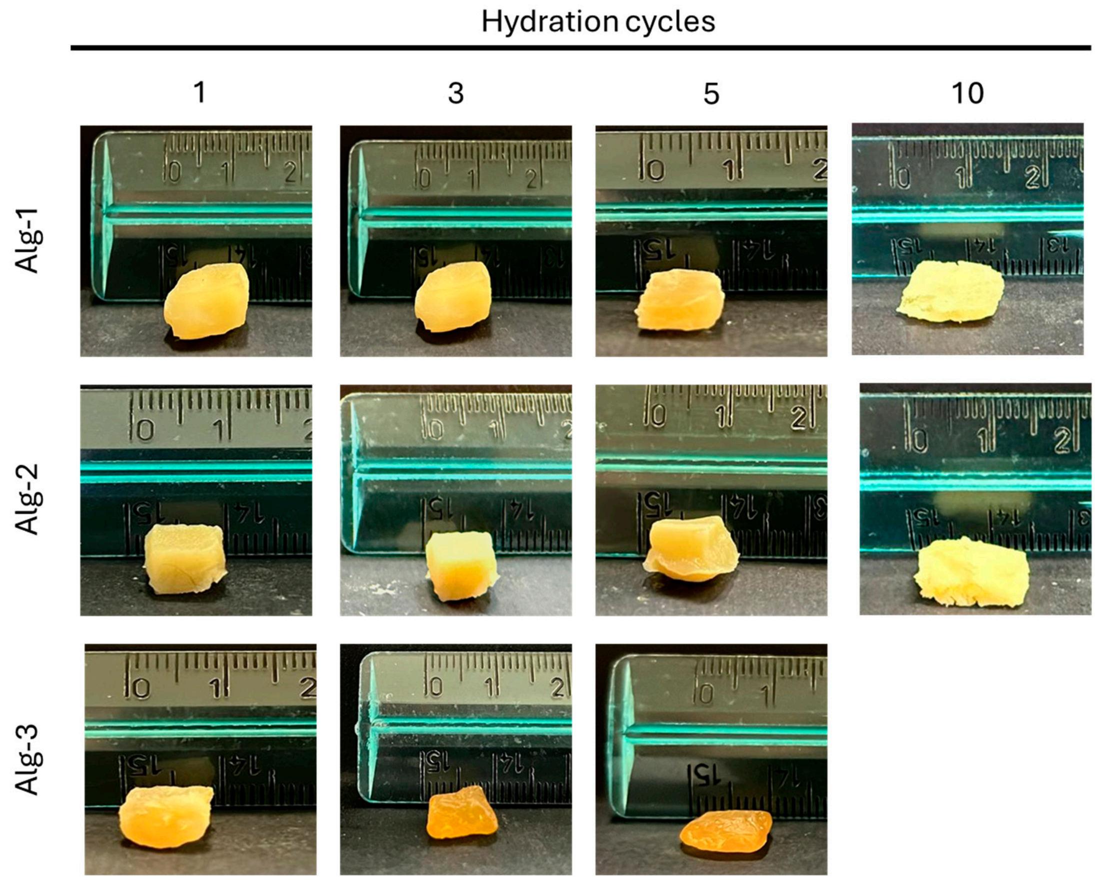

Figure S17. Visual appearance of the Alg-1, Alg-2, and Alg-3 polymer samples after 1, 3, 5, and 10 hydration-dehydration cycles. The images show the gels following the hydration phase of each cycle.

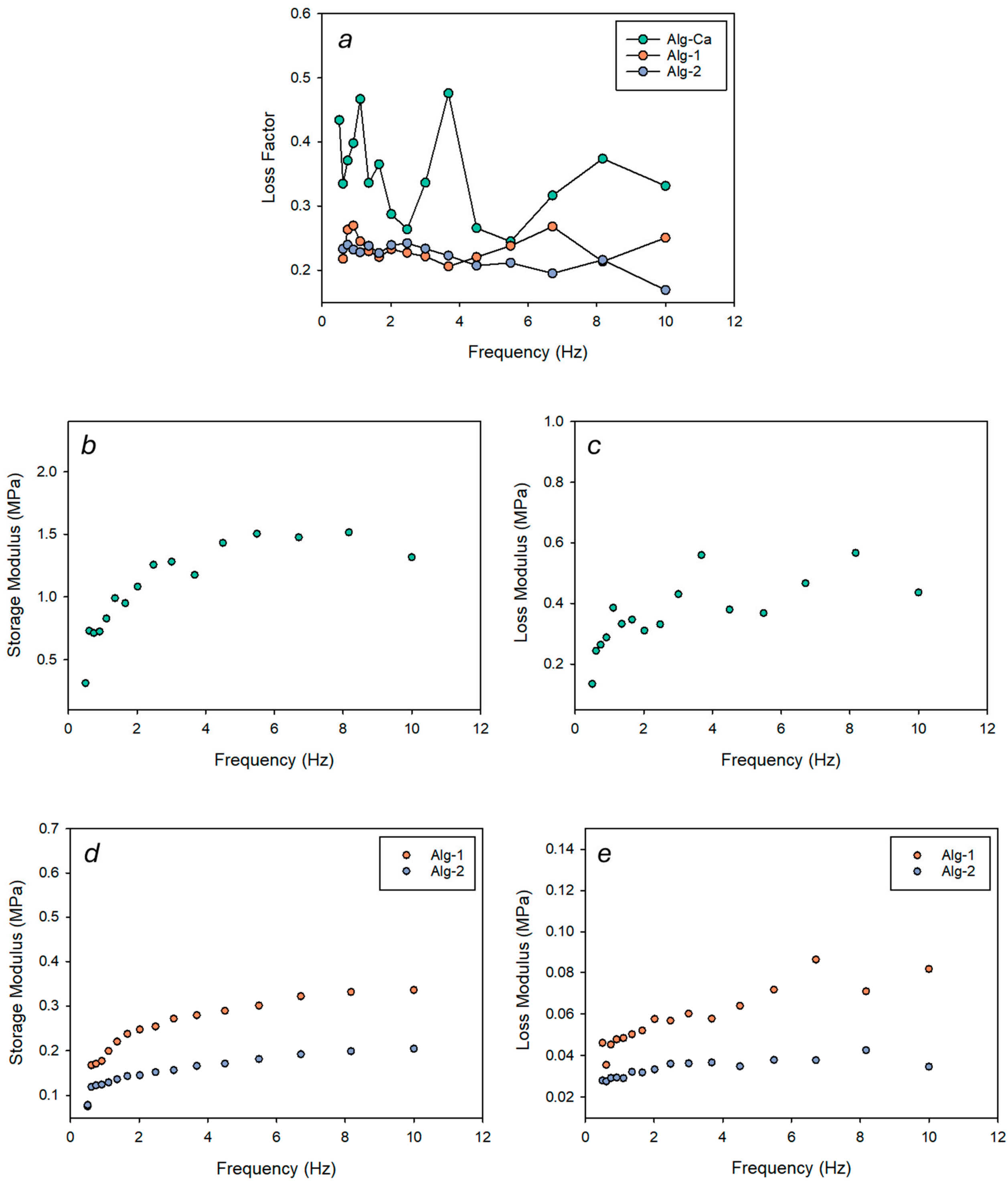

Figure S18. (a) Comparison of the loss factors for Alg-Ca, Alg-1, and Alg-2 gels; (b) storage modulus of the Alg-Ca; (c) loss modulus of the Alg-Ca; (d) storage modulus of Alg-1 and Alg-2; (e) loss modulus of Alg-1 and Alg-2.

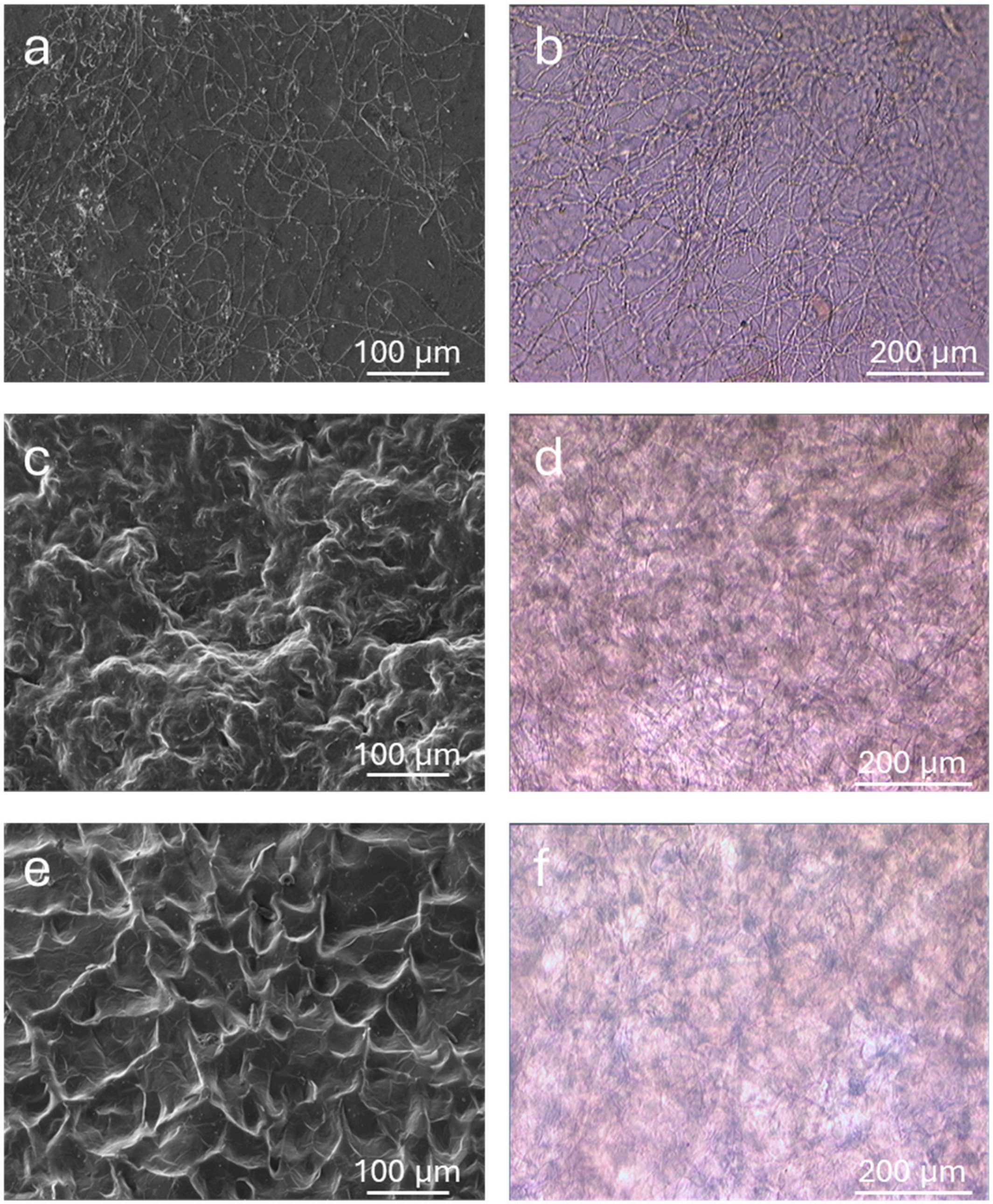

Figure S19. SEM and OM images of Alg-Ca, Alg-1, and Alg-2 hydrogel samples after antimicrobial assessment test: (a) Alg-Ca, SEM; (b) Alg-Ca OM; (c) Alg-1, SEM (d) Alg-1, OM; (e) Alg-2, SEM; (f) Alg-2, OM.

3. Preliminary application

This section reports the images related to the preliminary cleaning tests performed on the iron gall ink and wood samples.

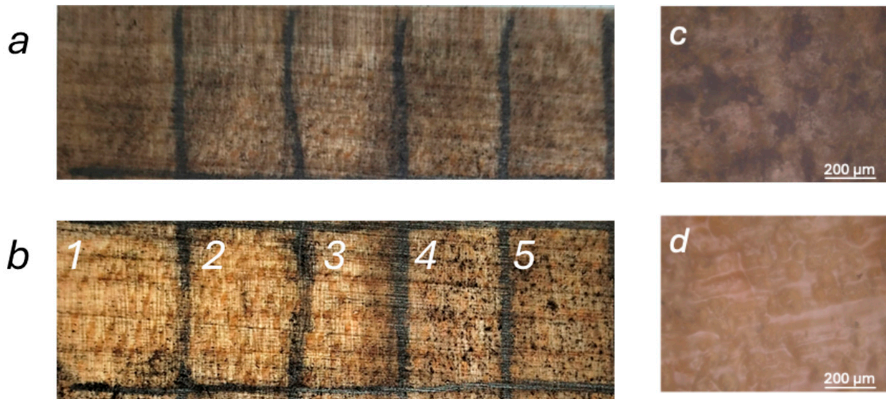

Figure S20. Picture of shellac-coated specimen: (a) after deposition of artificial dust; (b) after treatment with Alg-1 gel loaded with selected solvents: (1) 1%Ecosurf® in water; (2) 1-butanol/water 1:9; (3) ethanol/water 1:9; 4) nanoemulsion (9.7% 1-butanol, 20.9% butanone, and 3.5% Ecosurf® in water); 5) water; (c) optical microscope picture of the shellac-coated wood after soiling; (d) optical microscope picture taken after cleaning by Alg-1 loaded with 1%Ecosurf® in water.

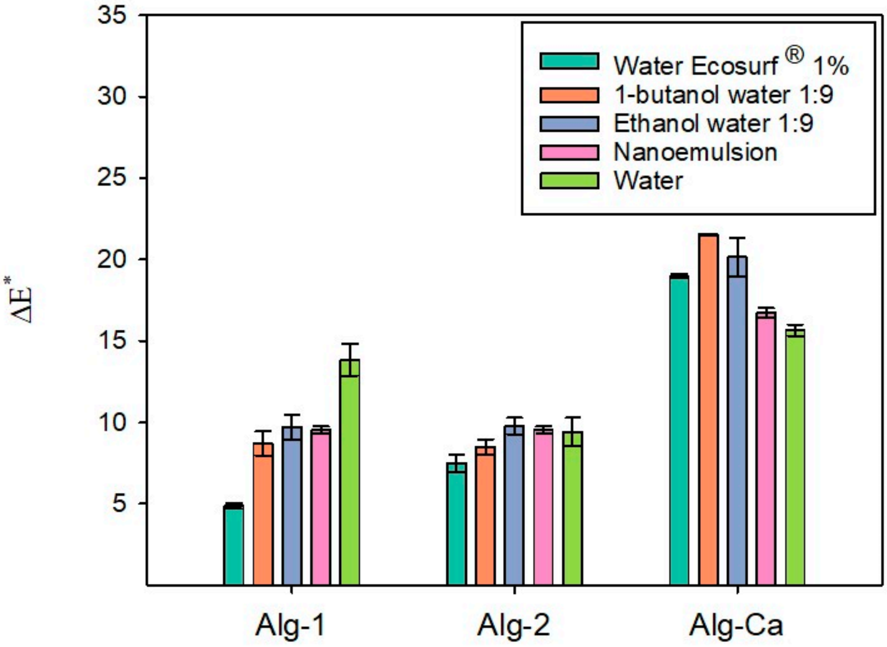

Figure S21. Colorimetric analysis of wood samples soiled with artificial dust. The bar chart reports the ΔE* values (with the respective standard deviation bars) after the cleaning process. Gels Alg-1 and Alg-2 were loaded with the following cleaning solutions: 1% Ecosurf® in water; 1-butanol/water 1:9 v/v; ethanol/water 1:9 v/v; nanoemulsion composed by 9.7% 1-butanol, 20.9% butanone, and 3.5% Ecosurf® in water; demineralized water.

Table S1. Colorimetric parameters (L*, a*, b*) of wood samples coated with shellac. The table reports the values obtained from three replicate measurements with the corresponding average values and standard deviations.

replicas	wood with shellac		
	L*	a*	b*
1	70.53	8.00	25.45
2	72.46	6.72	25.27
3	72.24	7.25	25.36
Average	71	7.3	25.4
Std.dev	1	0.6	0.1

Table S2. Colorimetric parameters (L*, a*, b*) of wood samples coated with shellac and soiled. The table reports the values obtained from three replicate measurements with the corresponding average values and standard deviations.

replicas	wood with shellac and soiled		
	L*	a*	b*
1	53.0	5.34	21.56
2	51.6	5.49	21.93
3	51.18	5.27	20.32
4	55.25	5.32	21.95
Average	53	5.4	21.4
Std.dev	2	0.1	0.8

Table S3. Colorimetric parameters (L*, a*, b*) measured on the wood surfaces after cleaning with different solvent mixtures (1% Ecosurf® in water; 1-butanol/water 1:9 v/v; ethanol/water 1:9 v/v; nanoemulsion composed by 9.7% 1-butanol, 20.9% butanone, and 3.5% Ecosurf® in water; demineralized water) and with the corresponding Alg-1, Alg-2, and Alg-Ca gels. For each test, three measurement points were analyzed on the surface, and the corresponding mean values and standard deviations are reported.

**Cleaning mixture**	**replicas**	**Gels**								
		**Alg-1**			**Alg-2**			**Alg-Ca**		
		L*	a*	b*	L*	a*	b*	L*	a*	b*
**Water Ecosurf® 1%**	1	70.54	7.55	30.11	67.15	7.88	31.22	52.22	6.14	23.40
	2	70.86	7.31	30.22	67.52	8.02	31.52	52.76	6.01	22.55
	3	70.95	7.78	30.01	66.89	7.63	31.02	53.97	5.38	22.36
	Average	70.8	7.6	30.1	67.2	7.8	31.3	53	5.8	22.8
	Std.dev	0.2	0.2	0.1	0.3	0.2	0.3	1	0.4	0.6
**1-butanol water 9:1**	1	63.62	7.06	27.42	67.92	8.70	32.60	51.41	5.59	21.72
	2	63.24	6.87	27.25	68.04	8.86	32.89	49.58	5.35	20.62
	3	62.91	6.65	26.98	68.15	9.05	33.06	51.00	5.53	21.56
	Average	63.3	6.9	27.2	68.0	8.9	32.9	51	5.5	21.3
	Std.dev	0.4	0.2	0.2	0.1	0.2	0.2	1	0.1	0.6
**Ethanol water 9:1**	1	63.25	7.62	30.20	64.88	8.57	32.07	54.26	6.32	23.58
	2	63.58	8.05	30.45	64.32	8.32	31.86	51.22	5.96	21.25
	3	63.41	7.85	30.26	65.12	8.92	32.31	50.19	5.62	21.73
	Average	63.4	7.8	30.3	64.8	8.6	32.1	51.9	6.0	22
	Std.dev	0.2	0.2	0.1	0.4	0.3	0.2	2.1	0.3	1
**Nanoemulsion**	1	71.22	9.15	34.69	64.58	8.67	32.12	54.28	5.49	23.04
	2	71.45	9.52	34.38	64.87	8.57	32.07	56.89	5.48	23.26
	3	70.82	8.75	35.02	64.41	8.38	31.97	54.74	5.28	22.63
	Average	71.2	9.1	34.7	64.6	8.5	32.1	55	5.4	23.0
	Std.dev	0.3	0.4	0.3	0.2	0.2	0.1	1	0.1	0.3
**Water**	1	57.93	6.28	24.97	63.05	7.29	28.95	56.22	5.71	23.89
	2	57.86	6.05	24.77	63.18	7.34	29.04	57.07	5.58	23.30
	3	58.05	6.38	25.18	62.89	7.22	28.78	55.66	5.57	22.90
	Average	57.9	6.2	25.0	63.0	7.28	28.9	56.3	5.62	23.4
	Std.dev	0.1	0.2	0.2	0.2	0.06	0.1	0.7	0.08	0.5

Table S4. ΔE* values measured on wood surfaces after cleaning with different solvents (solutions: 1% Ecosurf® in water; 1-butanol/water 1:9 v/v; ethanol/water 1:9 v/v; nanoemulsion composed by 9.7% 1-butanol, 20.9% butanone, and 3.5% Ecosurf® in water; demineralized water.) and with the corresponding Alg-1, Alg-2, and Alg-Ca gels.

Cleaning mixture	Alg1	Alg-2	Alg-Ca
Water Ecosurf® 1%	4.9 ± 0.2	7.5 ± 0.6	19.0 ± 0.1
1-butanol water 9:1	8.7 ± 0.7	8.5 ± 0.5	21.55 ± 0.04
Ethanol water 9:1	9.7 ± 0.8	9.8 ± 0.5	20 ± 1
Nanoemulsion	9.5 ± 0.2	9.9 ± 0.5	16.7 ± 0.3
Water	14 ± 1	9.4 ± 0.9	15.7 ± 0.4

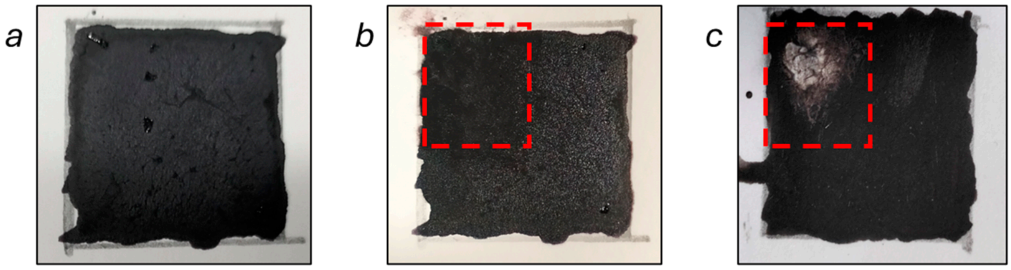

Figure S22. (a) Paper/Iron-gall ink mock-up before the gel application, (b) after the application of Alg-1 gel loaded with the ethanol/water 1:9 v/v solution with the treated area highlighted, and c) Paper/Iron-gall ink mock-up after the application of Alg-1 gel loaded with water, with the treated area highlighted.

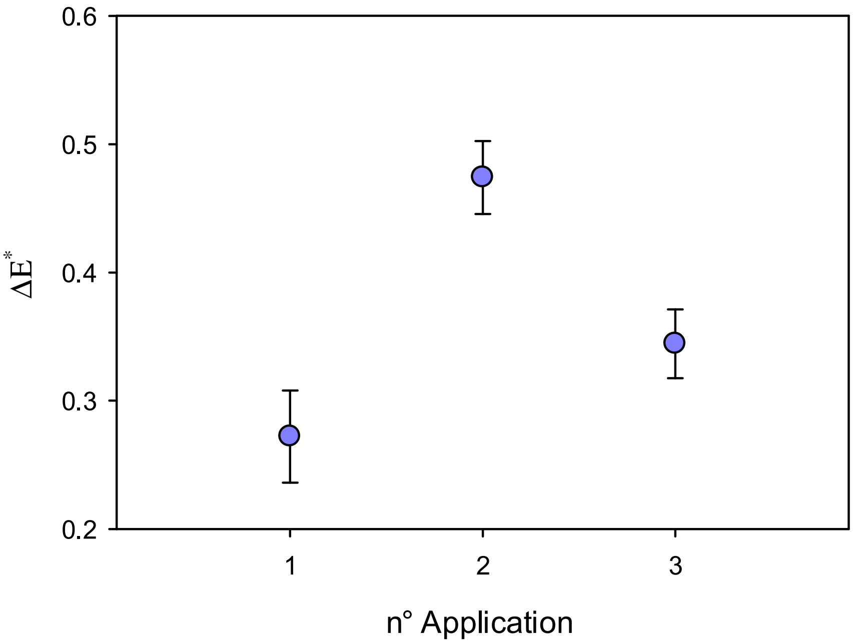

Figure S23. ΔE* values obtained by the colorimetric analysis of a paper/iron-gall ink after consecutive application of Alg-1 gel loaded with ethanol/water 1:9 v/v.

Table S5. Colorimetric parameters (L*, a*, b*) of the ink surface before artificial soil deposition, and after the cleaning cycles performed with the Alg-1 gel loaded with ethanol/water 1:9 v/v. The table also reports the corresponding ΔE* values calculated for each cleaning test.

**Analysis point**	**Before the application**			**After the application**											
				**1**				**2**				**3**			
	L*	a*	b*	L*	a*	b*	ΔE*	L*	a*	b*	ΔE*	L*	a*	b*	ΔE*
1	24.96	1.01	-3.73	25.06	1.12	-4.10		25.32	1.18	-4.16		25.21	1.14	-4.12	
2	26.07	1.19	-5.06	26.18	1.33	-5.18		26.33	1.42	-5.32		26.22	1.36	-5.20	
3	25.18	1.13	-5.55	25.37	1.21	-5.69		25.48	1.32	-5.77		25.42	1.24	-5.72	
Average	25.4	1.1	-4.8	25.5	1.2	-5.0	0.27	25.7	1.3	-5.1	0.47	25.6	1.3	-5.0	0.34
Std.dev	0.6	0.1	0.9	0.6	0.1	0.8	0.04	0.5	0.1	0.8	0.03	0.5	0.1	0.8	0.03

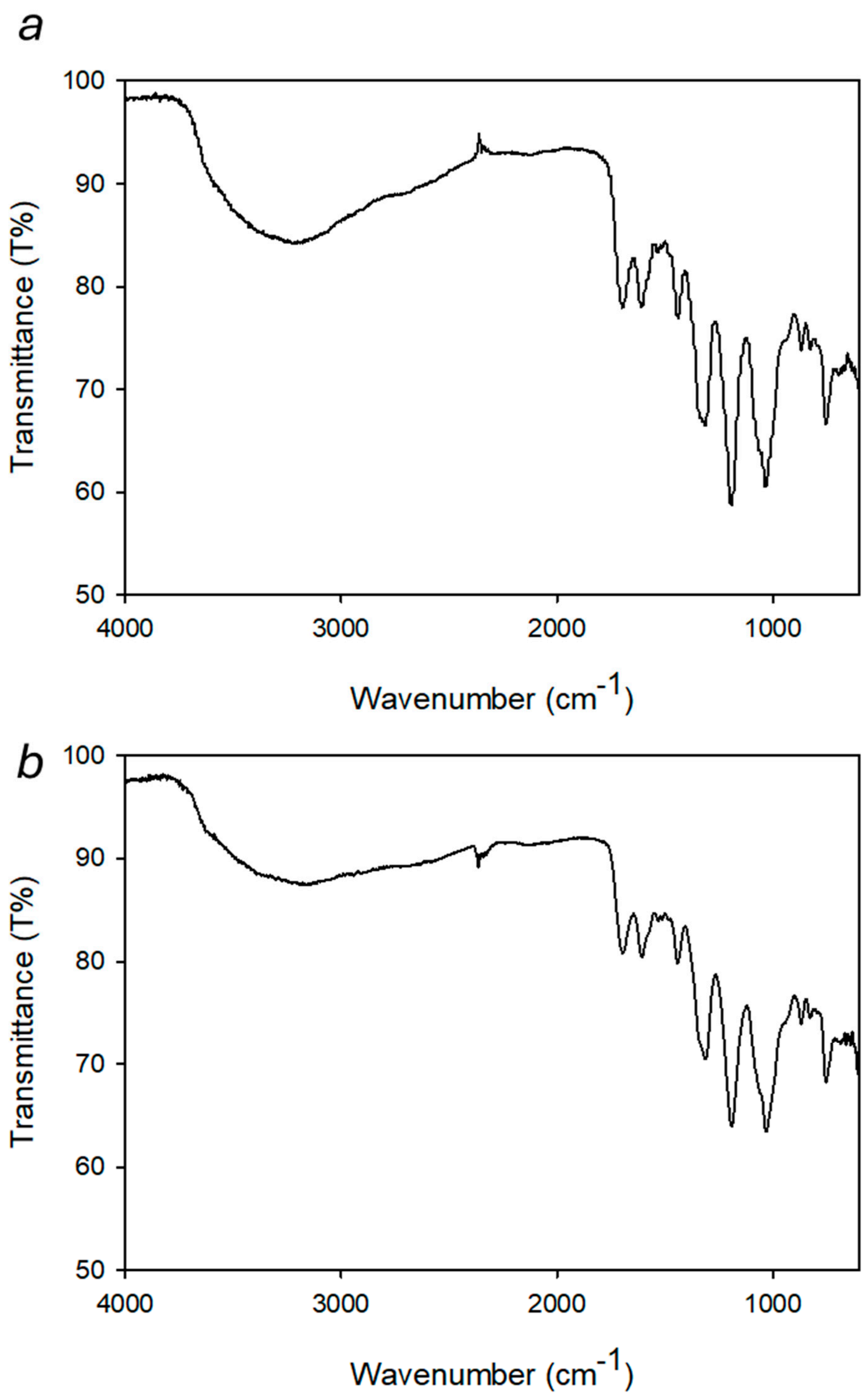

Figure S24. μ -FT-IR spectra of Iron-gall ink before (a) and after (b) the application of the gel Alg-1 with the solution ethanol/water 1:9 v/v.

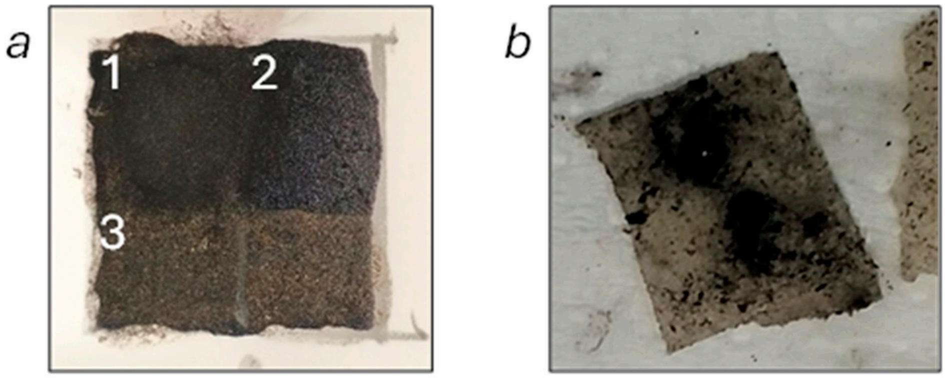

Figure S25. Mock-ups of iron gall ink: (a) after the cleaning procedure, showing area 1 cleaned from surface dust, area 2 as reference, and area 3 soiled with dust; (b) Alg-1 gel after the cleaning process.

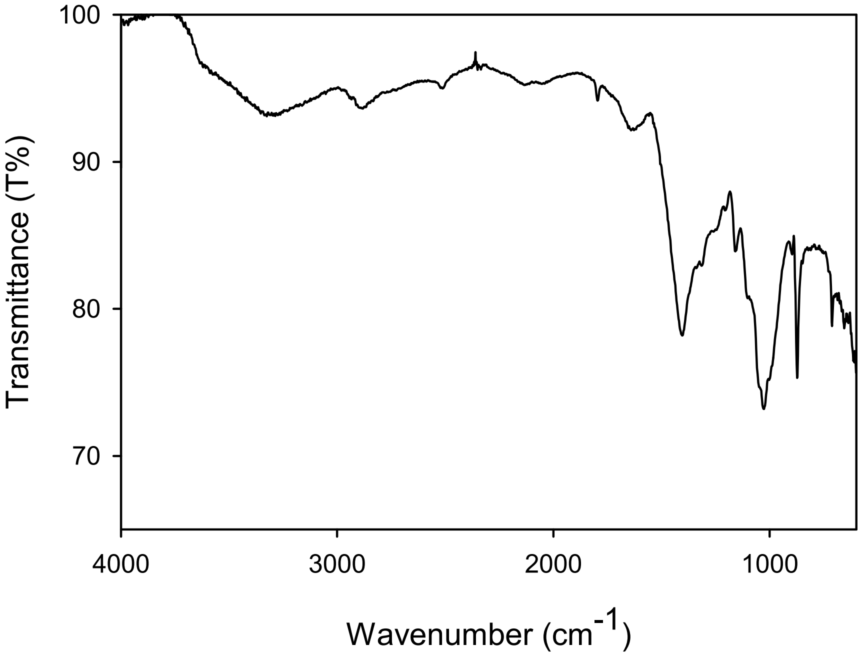

Figure S26. µ -FT-IR spectrum of Whatman 1 paper.

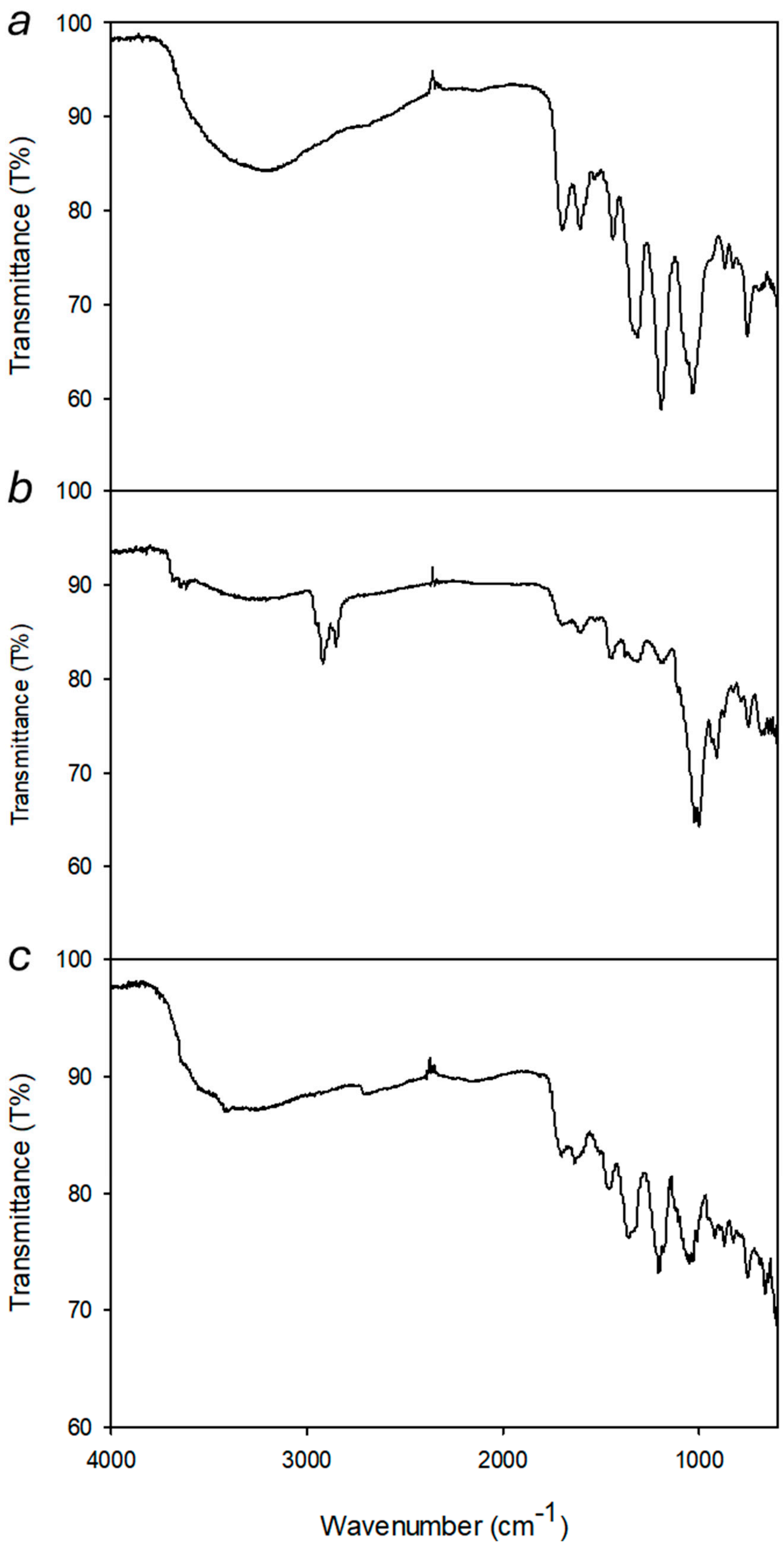

Figure S27. µ-FT-IR spectra of (a) paper with iron gall ink, (b) iron-gall ink on paper treated with artificial dust, and (c) paper-ink sample after the application of Alg-1 gel loaded with ethanol/water 1:9 v/v.
